# Cell-specific expression and individual function of prohormone convertase *PC1/3* in *Tribolium* larval growth highlights major evolutionary changes between beetle and fly neuroendocrine systems

**DOI:** 10.1186/s13227-021-00179-w

**Published:** 2021-06-29

**Authors:** Sonja Fritzsche, Vera S. Hunnekuhl

**Affiliations:** Johann-Friedrich-Blumenbach Institute, GZMB, Göttingen University, Göttingen, Germany

**Keywords:** *Tribolium castaneum*, Insect evolution, Prohormone convertases, *PC1/3*, *PC2*/*amontillado*, Larval development, Neuroendocrine system

## Abstract

**Background:**

The insect neuroendocrine system acts in the regulation of physiology, development and growth. Molecular evolution of this system hence has the potential to allow for major biological differences between insect groups. Two prohormone convertases, *PC1/3* and *PC2*, are found in animals and both function in the processing of neuropeptide precursors in the vertebrate neurosecretory pathway. Whereas *PC2*-function is conserved between the fly *Drosophila* and vertebrates*,* ancestral *PC1/3* was lost in the fly lineage and has not been functionally studied in any protostome.

**Results:**

In order to understand its original functions and the changes accompanying the gene loss in the fly, we investigated *PC1/3* and *PC2* expression and function in the beetle *Tribolium castaneum.* We found that *PC2* is broadly expressed in the nervous system, whereas surprisingly, *PC1/3* expression is restricted to specific cell groups in the posterior brain and suboesophageal ganglion. Both proteases have parallel but non-redundant functions in adult beetles’ viability and fertility. Female infertility following RNAi is caused by a failure to deposit sufficient yolk to the developing oocytes. Larval RNAi against *PC2* produced moulting defects where the larvae were not able to shed their old cuticle. This ecdysis phenotype was also observed in a small subset of PC1/3 knockdown larvae and was strongest in a double knockdown. Unexpectedly, most *PC1/3*-RNAi larvae showed strongly reduced growth, but went through larval moults despite minimal to zero weight gain.

**Conclusions:**

The cell type-specific expression of *PC1/3* and its essential requirement for larval growth highlight the important role of this gene within the insect neuroendocrine system. Genomic conservation in most insect groups suggests that it has a comparable individual function in other insects as well, which has been replaced by alternative mechanisms in flies.

**Supplementary Information:**

The online version contains supplementary material available at 10.1186/s13227-021-00179-w.

## Background

Signalling through neuropeptides represents an ancient pathway that is highly conserved between vertebrate and invertebrate taxa [1–3]. Many aspects of animal life such as life cycle, maturation processes, metabolism and behaviour are orchestrated by the neurosecretory pathway. Neuropeptides are commonly transcribed and translated as large precursor molecules (prohormones) that are, before secretion, post-translationally processed by specialised modifying enzymes. The genomic complement of orthologous genes encoding these enzymes is largely conserved, but notably, the well-studied but fast-evolving invertebrate model species *C. elegans* and *Drosophila* have suffered a loss of some neurosecretory pathway genes [1, 4, 5]. More specifically, subtilisin-like prohormone convertases of the PC-family cleave the precursor molecules at dibasic sites (often RR, KR) and are therefore central for the production of short bioactive peptides [6, 7]. Two of the subtilisin-like proteases, *PC1/3* and *PC2* are specific to the processing of neuropeptides. Both are expressed predominantly in the vertebrate central nervous system and neuroendocrine glands. Structurally they contain an active proteolytic site and an N-terminal signal peptide directing them to the rough ER and ultimately to the secretory vesicles. It is believed that all neuropeptides containing dibasic sites are targeted by either *PC1/3*, *PC2* or both*.* Mouse mutants show multiple, partially overlapping defects in neuropeptide production and null mutations display increased lethality (*PC1/3*) and reduced growth (*PC1/3* and *PC2*) (reviewed in [7]).

As in vertebrates, insect neuropeptide signalling regulates essential biological processes such as behaviour, feeding and physiology, water balance, as well as life cycle transitions and reproduction [8, 9]. The neuroendocrine function of *PC2* is conserved between mouse and *Drosophila*. Mutations of the fly’s *PC2* orthologue *amontillado (amon)* lead to impaired production of multiple neuropeptides and is lethal [6, 10–12]. However, the fly is a rather atypical model for PC-function as it has lost the ancestral *PC1/3* gene [6, 10], while both PCs are present in other insect genomes [1, 13]. Hence, work in the fly is unlikely to reveal a comprehensive picture of ancestral PC-function and additional studies on alternative insect models are required. This is particularly interesting because PC-processing-dependent neuropeptide signalling is thought to be involved in a multitude of biological processes. These include for example metabolic regulation by insulin-like peptides and adipokinetic hormone [14, 15]. Insulin, among other peptides, is involved in the nutrient-dependent regulation of oocyte maturation [16]. In addition, neuropeptides are involved in the regulation of insect-specific evolutionary inventions like their growth and development through distinct larval and pupal stages separated by moults [17, 18]. The mutant phenotype of the fly *PC2* gene *amon* is developmental arrest during the first instar larval moult when the animal is not able to shed its old cuticle [12]. However, nothing is known on PC-function in moulting and other processes in any other insect but the fly, and in particular, there is no functional information on *PC1/3* function as this gene was lost in flies. One hypothesis would be that *PC2* and *PC1/3* in other insects act in a near-redundant fashion and that *PC2* would be able to largely compensate for loss of *PC1/3*, explaining how the loss of *PC1/3* could have occurred in the fly lineage. In an opposing scenario, *PC1/3* may have specific functions in other insects, which have become unnecessary in the fly due to changes in lifestyle or alterations in the regulatory pathways.

Here, we investigate *PC2* and *PC1/3* gene function in the beetle *Tribolium castaneum* in order to study the functional evolution of neuroendocrine processing by comparing the well-known but derived *Drosophila* situation to an insect with a conserved complement of these enzymes and a different lifestyle. *Tribolium castaneum* is an alternative insect model system which is amenable to functional genetic work [19, 20] and imaging of the CNS [21]. Furthermore, *Tribolium* has already been subject of some studies looking at the function of individual neuropeptides [22, 23].

First, we confirmed that *PC1/3*, which is evolutionarily older than *PC2*, is conserved in all insect groups except for dipterans. This genomic conservation is suggestive of a functional importance of retaining separate *PC1/3* and *PC2* orthologues. Indeed, we demonstrate that both *Tribolium PC2* and *PC1/3* have an essential function in maintaining metabolic balance and nutrient-dependent fertility in adult beetles. In larvae, we found that the role of *PC2* in larval-to-larval moulting is conserved between fly and beetle producing an *amontillado*-like ecdysis phenotype. We also find that *PC1/3* backs up *PC2* function in the moulting process as a double knockdown increases the penetrance of the ecdysis phenotype. Unexpectedly, we characterise a novel and important individual role of *PC1/3* at the larval stage: the gene is expressed in small cell groups of the CNS and is essentially required for larval growth. Interestingly, despite the lack of growth, moulting is not inhibited in these RNAi-larvae.

In conclusion, the parallel functions in adult beetles are comparable to the overlapping but not fully redundant functions of both these genes in vertebrates [7], whereas they both have additional insect-specific functions at the larval stage. We discuss our findings on *PC1/3* gene function in connection with current models on the interconnection of larval-to-larval moults with weight gain [17, 24].

## Results

### *PC1/3* has a pre-bilaterian origin and is conserved in all insect groups except flies

**Fig. 1:**
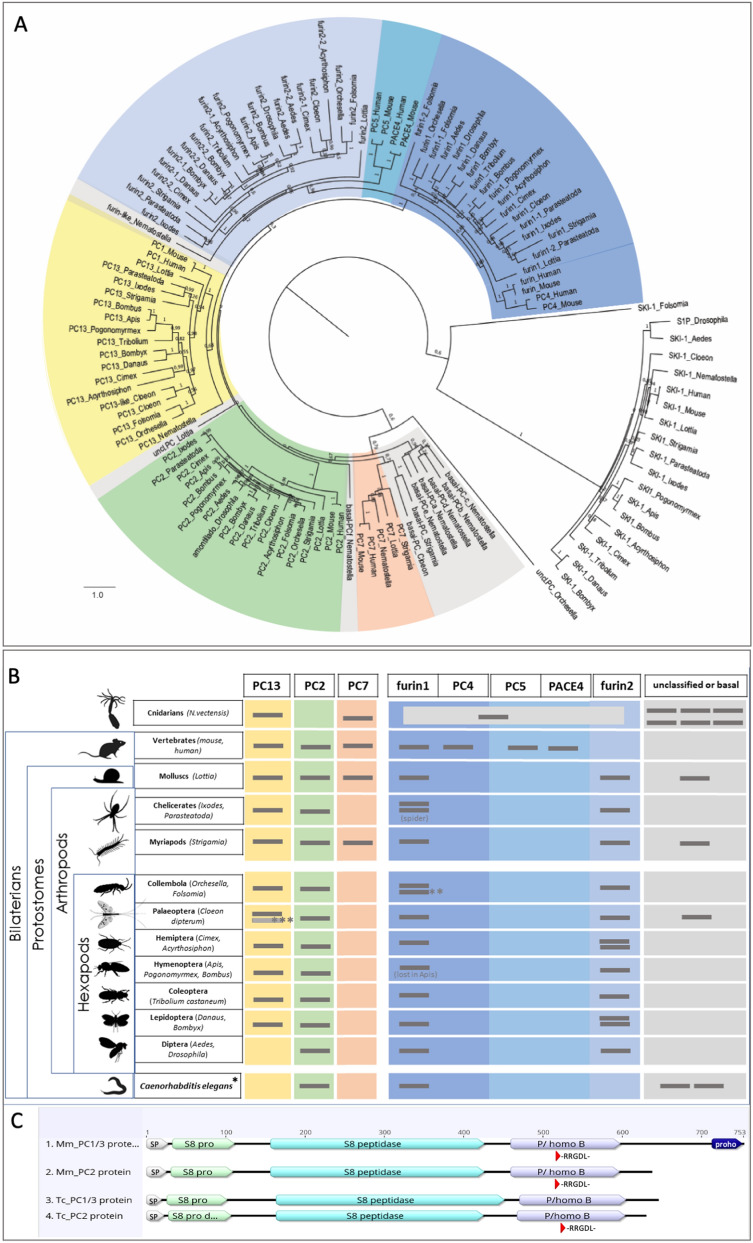
Genomic evolution of subtilisin-like prohormone convertases. **A** Gene tree (based on protein sequence retrieved from Genbank nr database and ENSMBL proteins between 06-2020 and 12-2020) showing the clustering of prohormone convertases and furins in different animal groups, including most major insect taxa. SKI-1-proteins were used as outgroup. Node labels indicate local bootstrap support values. Full species names and protein sequences used in the alignment are given in Additional file 1: Table S1. **B** Table showing presence/absence distribution of PC-orthologues in insects and other animal groups (*classification of *C. elegans* proteins is based on [4]). Overall, the system is conserved. A *PC1/3* but no *PC2* orthologue is present in cnidarians, identifying *PC1/3* as the evolutionarily older convertase. Only dipterans and *C. elegans* have lost *PC1/3*. All insects have lost the *PC7* genes, whereas it was retained in the myriapod *Strigamia maritima* and the lophotrochozoan *Lottia gigantea.* A group of basal PCs closely related to *PC7* genes is present in the Cnidarian *Nematostella*. The myriapod *Strigamia maritima* and the mayfly *Cloeon dipterum* have sequences that appear to be closely related to PC7 proteins and pre-bilaterian basal PCs. The bilaterian invertebrate species each have *furin1* and *furin2*, whereas only 1 furin protein is present in the cnidarians. Vertebrate furins have diverged into *furin* and *PC4* (related to invertebrate *furin2*) as well as *PC5* and *PACE4* (related to invertebrate *furin1*). **The *Orchesella cincta* sequence (uncl.PC_*Orchesella*, see tree in **A** does not group with any of the subgroups. Based on the presence of a second *furin1* gene in the other included collembolan *Folsomia candida* (furin1-2_*Folsomia*) we assume that the *Orchesella* sequence might be a derived *furin1* duplicate, even though this is not supported by the alignment). *** A second *Chloeon* PC1/3 sequence might be an assembly artefact (see suppl. material 1). All cartoons credited to PhyloPic/free licence, hemipteran: K. Garcia/PhyloPic. **C** Protein structure of *Tribolium* PC1/3 and PC2 in comparison to vertebrate (mouse) PC1/3 and PC2, showing the N-terminal Signal Peptide (SP), S8-pro domain, S8-peptidase domain, the P/homo B regulatory domain, and a prohormone domain (proho) present in mouse PC2. A specific sequence motif including -RRGDL- conserved in vertebrates and possibly involved in the sorting of the proteins to secretory vesicles [27] is present in *Tribolium* PC2 but not PC1/3

To understand the evolutionary history of the insect subtilisin-like protein convertases and to map patterns of gene loss, we scored genomes of selected species for presence or absence of these enzymes. There are seven PC-proteins in mice and humans (PC1/3, PC2, PC5, PC4, PC6/PACE4, PC7 and furin), in addition to PCSK-9 and SKI-1, which also contain subtilisin-like catalytic domains but are membrane bound [25]. We surveyed well-assembled genomes representing major insect lineages in addition to other animal lineages. We then used identified PC protein sequences for gene family reconstruction (see gene tree Fig. 1A), using SKI-1 proteins as an outgroup (PCSK-9 was not found in any invertebrate genome).

When comparing different bilaterians it became evident that a set of four PCs is shared between disparate groups: *PC1/3*, *PC2*, *PC7* and *furin* (see Fig. 1A, B). These were found in representatives of arthropods, molluscs and vertebrates, representing the major bilaterian lineages. *Nematostella vectensis*, belonging to the basally branching radial symmetric cnidarians, has a clear *PC1/3* and a *PC7* orthologue, and one gene that groups with *furins*, but lacks *PC2*. In addition, there are six cnidarian PCs that do not group with any of the bilaterian convertases but form a group of their own (Fig. 1A). The vertebrate furin proteins have undergone an expansion. Vertebrate *PC4* and *furin* are closely related and both cluster with invertebrate *furin1*. The PC5 and PACE4 proteins cluster with invertebrate furin2 and are closely related to one another (Fig. 1A).

When compared to vertebrates, the PC protein family is more compact in insects, typically comprising PC1/3, PC2, furin1 and furin2 (with some additional lineage specific duplications of furin genes in hemipterans, lepidopterans and collembolans Fig. 1A, B). Two *furin* genes are the typical gene content in invertebrates as direct orthologues to the insect *furins* are present in the other invertebrate taxa such as chelicerates, myriapods and the lophotrochozoan *Lottia gigantea*. In the myriapod *Strigamia maritima*, an arthropod with a conservative genome organisation [26] a *PC7* gene is present. It was lost, presumably independently, from spiders and all hexapod genomes. By contrast *PC1/3* is conserved in all insect groups, but no orthologues were detected in the dipteran genomes that we sampled (*Drosophila melanogaster* and *Aedes aegypti*). In addition, BLAST searches for *PC1/3* genes in the entire dipteran clade did not identify any orthologous sequence (last verified in 05–2021). Hence the factor was specifically lost in the lineage leading to the Diptera. Similar to flies, *Caenorhabditis elegans,* the other major invertebrate model organism, has lost its *PC1/3* orthologue but only possesses a clear *PC2* orthologue [4].

To score the degree of conservation of the *Tribolium* prohormone convertases, we compared the protein sequences. On the protein level the *Tribolium* neuroendocrine specific convertases PC1/3 and PC2 share moderate overall sequence similarity of 44%. However, they have a near identical organisation. An N-terminal signal peptide that directs the polypeptides to the neurosecretory pathway is followed by an S8-pro- and S8-peptidase catalytic domain exerting the enzymatic functions, and a P/homo B regulatory domain (see Fig. 1C). This domain structure is conserved between the *Tribolium* and vertebrate neuroendocrine specific PCs [7]. A specific amino acid motif with a core of -RGD- has been identified within the active domain of vertebrate PC1/3 and PC2 and was shown to be required for sorting the vertebrate enzymes into the secretory vesicles [27]. This motif is present in *Tribolium* PC2 and in all other insect PC2 proteins that we have looked at. However, the motif is absent from the PC1/3 protein of *Tribolium* and other insects, and from the myriapod *Strigamia*, but present in the cnidarian and chelicerate PC1/3 proteins.

### *PC2* is expressed ubiquitously in cells of the nervous system, whereas *PC1/3* is restricted to identifiable cell populations

*PC1/3* has not been studied in any insect/arthropod before. Given that *Tribolium* contains the arthropod-typical set of two PCs, we asked, whether *PC1/3* and *PC2* were expressed in different or overlapping cell groups. We performed RNA in situ hybridisation to detect specific expression of these genes in embryos and in the larval nervous system of *Tribolium.* Limited sequence similarity between the paralogues on DNA-level allowed for the production of specific RNA-probes (see Additional file 1: Figure S1).

**Fig. 2:**
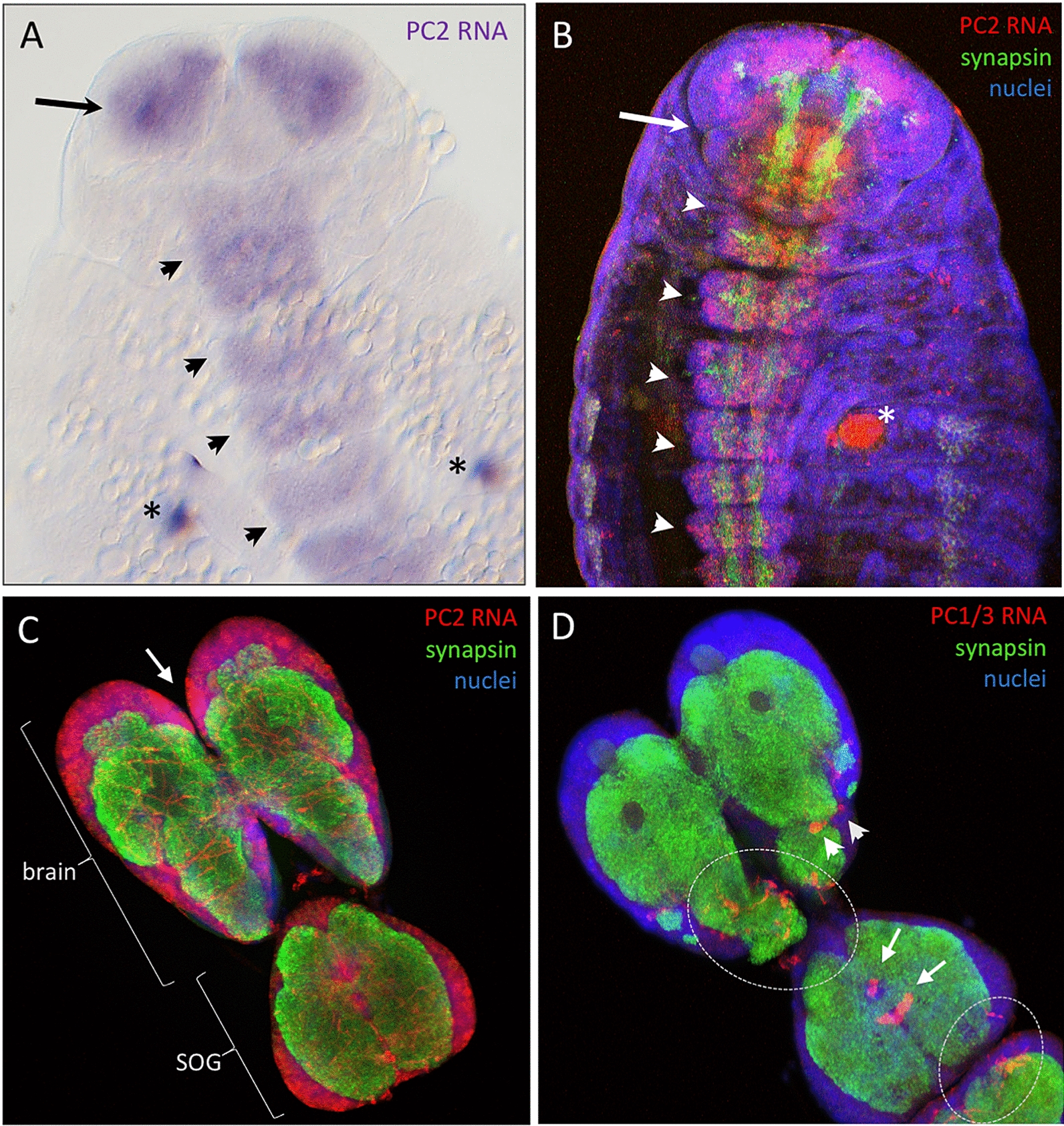
Divergent expression of *PC1/3* and *PC2* in *Tribolium*. Stages according to [28], NS14 is the last germband stage in which the legs reach the anterior tips of the posteriorly following segment after the next, stage 15 is the last embryonic stage before hatching when the whole embryo is of an oval shape. **A** NBT/B-ZIP colorimetric stain of embryonic *PC2* expression, showing the anterior half of an embryo of stage NS14. The signal is located to the ventral neuroectoderm (arrowheads) and prospective brain area (arrow). **B** Fluorescent in situ stain of an embryo at stage NS15. *PC2* expressing cells (red) are associated with the developing central nervous system in the ventral neuroectoderm (arrowheads) and the prospective brain (arrow). Note that not all neurons are visible in this projection: the anterior brain commissures are already present but are located on a deeper level. The strong staining in the first abdominal segment (seen in **A** and **B**, asterisks) represents unspecific signal from the pleuropods. **C** Ubiquitous *PC2* expression in the anterior larval nervous system. Shown is the brain and suboesophageal ganglion (SOG). Cell bodies and nuclei are located in the periphery and project their axons into the central neuropile (visualised by anti-synapsin). Some more intense staining is visible in the anterior medial brain (arrow). **D** *PC1/3* expression in the larval nervous system is restricted to individual cells of the dorsal side of the suboesophageal ganglion (arrows) and the posterior brain (arrowheads). Those cells are present bilaterally, but signal is obscured on the left side by unspecific signal of trachea (encircled areas). **B**/**C**/**D** are maximum intensity Z-projections (created with imageJ) and are based on multichannel confocal laser scanning image stacks with each channel using the same plane thickness to avoid optical shift

We found that *PC2* is expressed during late embryogenesis (NS 14, which is still a flat germband with the tips of the legs reaching the anterior part of the second posteriorly following segment [28]) in many cells of the head and trunk neuroectoderm (Fig. 2A). At a slightly later stage (NS 15, shortly before hatching when the whole embryo is of oval shape [28]) most neural cells of the developing central nervous system express *PC2* (Fig. 2B). At the larval stage, *PC2* expression is found in most cells of the nervous system (Fig. 2C). A more intense expression is seen in the anterior medial brain area in larvae (arrow in Fig. 2C) where the *pars intercerebralis* is located, a structure of the insect brain with known neuropeptidergic properties [29].

By contrast, we did not find any embryonic expression of *PC1/3* (see Additional file 1: Figure S2). Strikingly, expression in the larval nervous system was restricted to individual cells. In the suboesophageal ganglion we found two dorsally located cell groups. The anterior cluster comprised two cells (white arrow in Fig. 2D) and the more posterior normally contained 6 cells (white arrow in Fig. 2D). In one of four inspected nervous systems the posterior cluster comprised 8 cells, indicating some variability. Expression was also found laterally in the posterior brain (tritocerebrum) where two cells on each side stain positive (arrowheads Fig. 2D). We found no signal in the PI or other cells of the protocerebrum, nor in the mid-thoracic ganglia. With the methods available to us, we were not able to screen for *PC1/3* or *PC2* expression in the peripheral nervous system or neuroendocrine glands.

### Divergent functions of *PC1/3* and *PC2* in larval growth

Given that most arthropods have both *PC1/3* and *PC2* and given the different expression patterns in *Tribolium* we hypothesised that these enzymes would have important and at least partially different functions in larvae. To this end we used larval RNAi to test for effects of *PC1/3* and *PC2* gene knockdown on larval development. The use of two non-overlapping fragments produced similar effects, indicating that the phenotypes were specific (also see Additional file 1: Figure S1 for a DNA sequence alignment and annotation of the dsRNA fragments, and see Additional file 1: Figure S3 for quantification of knockdown by qPCR).

We first injected dsRNA targeting the respective genes into 12-day-old L4 larvae. At 32 °C *Tribolium* undergoes 6 larval moults (L1–L7) and larval stage 4 normally starts from day 11 after egg lay, although there is some natural variation within this timing. On day 12 80% of larvae had completed the 3^rd^ larval moult to L4 and no larvae were at stage L5 by then. We hand-picked average-sized larvae to exclude differing stages from the experiments.

We also included a double knockdown of both factors in our analysis. Growth curves and moult cycles were recorded following the injections.

**Fig. 3:**
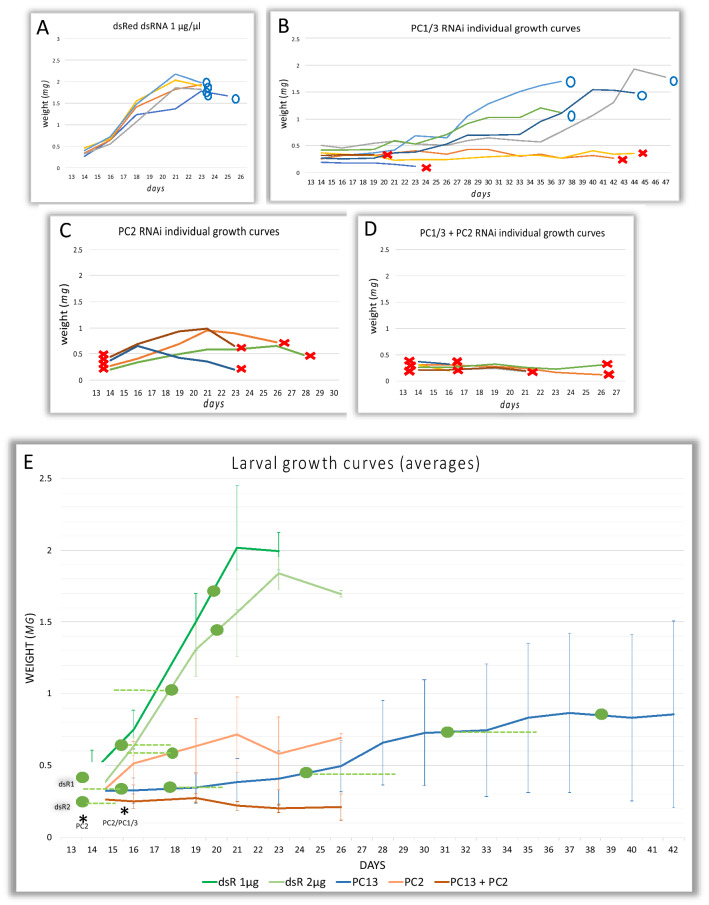
*PC1/3* and *PC2* RNAi affect larval growth and survival. All larvae were injected at 12 days after egg lay (4^th^ larval instar) at a concentration of 1 µg/µl unless stated otherwise. Blue circles indicate successful metamorphosis to pupae, red crosses mark deaths. **A** Growth curves of individual larvae injected with dsRed-dsRNA (*n* = 5). Larvae undergo constant growth until a weight of app. 2 mg is reached. Larvae lose weight during the pre-pupal stage and pupate between day 21 and 23 of development. One larva with transiently reduced growth pupated later but at a similar weight. These results were similar to untreated wild-type larvae (see Additional file 1: Figure S4). **B** *PC1/3*-RNAi larvae (*n* = 8) show strongly reduced growth and 50% die prematurely. The other half reach the pupal stage with strong delay (all surviving spent more than 35 days at the larval stage). **C** *PC2*-RNAi larvae (*n* = 8) initially show a better growth than *PC1/3*-larvae but then all suffer severe weight loss and die as larvae. Lethality is 100%. **D** Double *PC1/3*- and *PC2*-RNAi (1 µg/µl each) (*n* = 8) leads to a combined phenotype with no weight gain and early death. Total injected dsRNA concentration was 2 µg/µl, see Additional file 1: Figure S4. **E** Average growth curves of each RNAi treatment based on data shown in **A-D** and including a *dsRed* control injected at 2 µg/µl, matching the total dsRNA concentration of D (individual curves not shown). Error bars indicate standard deviations. Green circles indicate the time interval between two data points in which > 50% of larvae completed a moult. Dashed lines mark the interval by which the remaining (surviving) larvae completed the moult (line going to the right means additional moults occurred after the main data point, line going to left indicates additional moults have occurred before). Green circles marked with dsR1 and dsR2 on day 13 indicate that > 50% of *dsRed* 1 µg/µl- and 2 µg/µl-injected larvae underwent a moult between injection and first data acquisition. Black asterisks indicate that a small proportion (< 50%) of the *PC2*-RNAi larvae (1/8) and of the *PC1/3*-PC2-RNAi larvae (3/8) underwent a moult in the respective time intervals. *PC1/3*-RNAi larvae undergo supernumerary moults while showing severely reduced weight gain. Graph only includes development to day 42, after which no more moults occurred. Individual data of *PC1/3* larval growth and moult cycle is given in Additional file 1: Table S4

Wild-type and *dsRed* dsRNA-injected control animals showed a constant weight gain until they reached a weight of over 2 mg (Fig. 3A and see Additional file 1: Figure S4 for growth curves of untreated and *dsRed* dsRNA control-injected larvae). Then the larvae lost some weight as they stopped feeding in preparation for the larva to pupa transition (Fig. 3A, S4A). By contrast, *PC1/3* RNAi-treated larvae stopped growth almost completely for several days (Fig. 3B). Some of the observed larvae showed near zero weight gain over a period of up to 30 days, and some of these individuals died prematurely (larval lethality was 50%). Another half of the larvae did initially show a flat growth curve but entered a phase of increased weight gain later, presumably when the RNAi-effect had faded out over time, and eventually reached a weight sufficient for larva to pupa transition. Due to the slowed growth the larval period was significantly prolonged in *PC1/3* knockdown larvae: all control larvae had pupated by day 28 of development whereas 50% of *PC1/3*-RNAi specimens remained in the larval period beyond the age of 41 days. In an independent experiment, we observed an individual *PC1/3*-dsRNA-treated animal that had not pupated even after 3 months’ time. Intriguingly, we observed supernumerary moults in the knockdown larvae (see below).

*PC2* dsRNA-injected larvae had a high mortality and half of them had already died on day one after injection (Fig. 3C), compared to zero deaths in control-injected larvae at this timepoint. Unlike *PC1/3* knockdown larvae *PC2*-RNAi larvae initially showed some weight gain, but still lagged behind the control sets (see Fig. 3C, E). All *PC2*-RNAi larvae eventually suffered rapid weight loss and were found dead shortly after. Larvae that were co-injected with *PC1/3*- and *PC2*-dsRNA, each at the same concentration of 1 µg/µl dsRNA targeting each gene, showed a strongly reduced growth as known from the *PC1/3* knockdown and a high lethality as observed in *PC2* larvae (Fig. 3D) indicating additive function. Results of independent repeat experiments confirming the effect of *PC1/3* and *PC2* knockdown in larvae are shown in Additional file 1: Figure S5.

In conclusion, these results showed that *PC1/3* and *PC2* are both required to maintain larval growth and survival. The different dynamics, however, indicated that both function independently at the larval stage.

### PC1/3 knockdown leads to the decoupling of growth from moulting

Under normal conditions wild-type *Tribolium castaneum* undergo 6 larval moults and the timing of larval moulting is thought to depend on the growth rate. We wondered how the reduced growth in *PC1/3* and *PC2* knockdown would affect the number of moults. Therefore, we plotted the moults on the average growth curves based on the above-described experiments (Fig. 3E, green circles indicate the point in time when most animals moulted, the green dotted line marks the time range when moults of the remaining larvae occurred). Most (5/8) *PC2* injected larvae underwent one moult after some weight gain, but accompanied by the high lethality, the majority of these larvae (4/5) did not go through the second moult cycle. Only a small proportion of *PC1/3* and *PC2* double RNAi animals did complete one moult (3/8), and none of them completed a second moult (Fig. 3E). Unexpectedly, larvae treated with only *PC1/3* dsRNA still went through larval moults despite strongly reduced growth (see above). They underwent the first two moults at similar points in time compared to control injections although there was almost no weight gain during that period (Fig. 3E). Some individuals went through moults without effective weight gain since the last moult (see Additional file 1: Table S4). The third moult was delayed by more than four days compared to the last larval-larval moult of the wild type. Interestingly, the *PC1/3* knockdown larvae went through supernumerary moults while the increase in weight was minimal.

Taken together, our experiments showed that *PC1/3*-knockdown led to a strong inhibition of larval growth and to the decoupling of larval moults from the growth process.

### *Amontillado-*like ecdysis phenotypes occur at a high penetrance in *PC2* knockdown, and at a low frequency, in *PC1/3* knockdown larvae

**Fig. 4:**
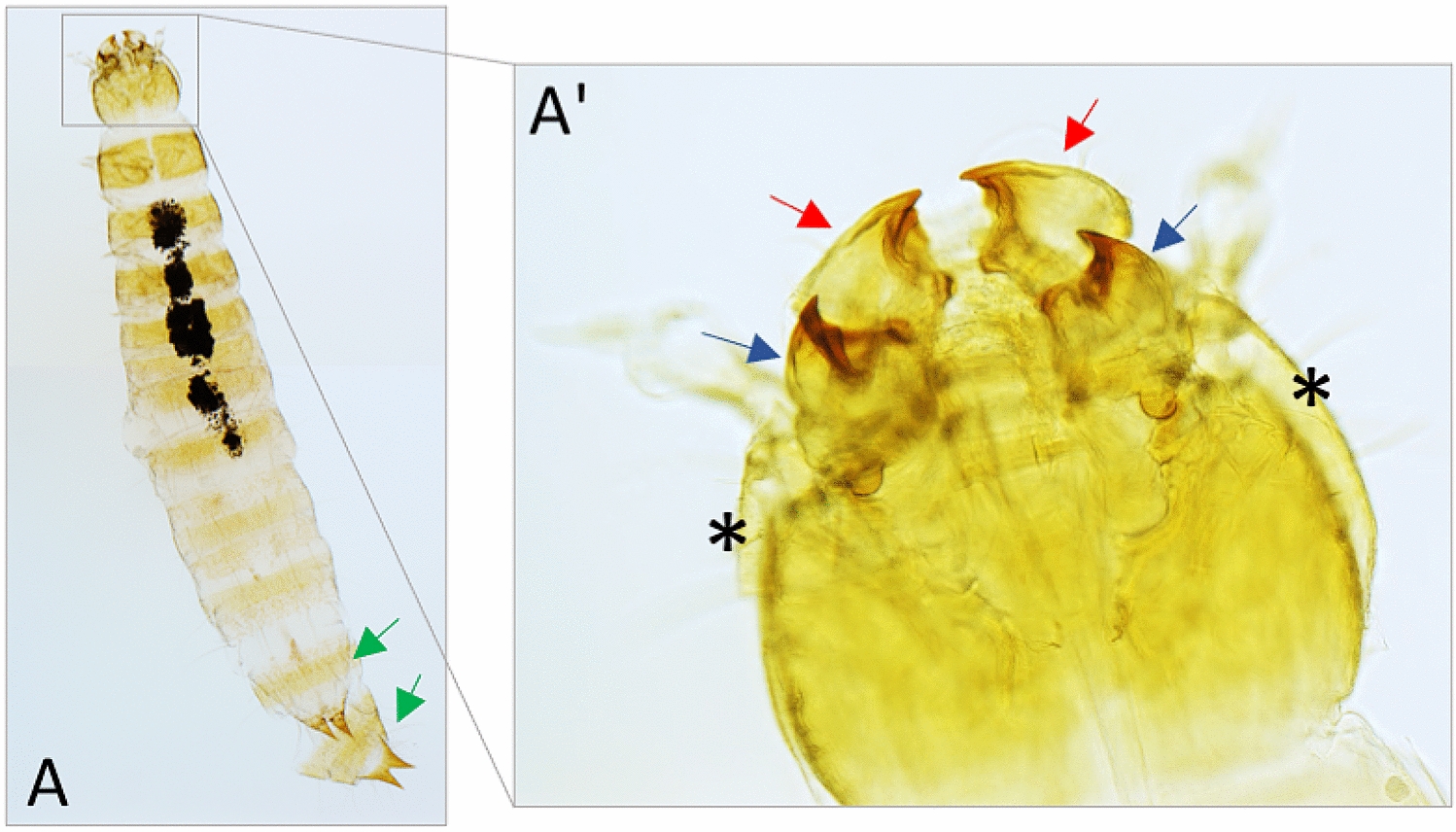
*Amontillado*-like ecdysis phenotypes following larval RNAi against *PC2*. **A/A’** Cuticle preparations reveal duplicated cuticular structures like the terminal cerci (green arrows in **A**) and mandibles (red and blue arrows in **A’**). The second cuticle is also visible laterally on the head (black asterisks in **A’**) Specimen shown was treated with *PC2*-RNAi, frequencies of this phenotype across different knockdowns are given in Table 1

Mutants of the *Drosophila PC2* orthologue *amontillado* show a failure of the moulting process: the larva produces a new cuticle but is not able to shed its old cuticle and becomes “locked-in”, which then leads to weight loss and death [12]. Hence, *Tribolium PC2*-RNAi larvae were specifically screened for this phenotype once they had died. Indeed, a second cuticle was detected, unambiguously recognisable by the duplicated mandibles and terminal structures (Fig. 4A, A’).

By screening a number of dead *PC2* knockdown larvae (injected at 12 days) we found, that 75% (*n* = 20) (see Table 1) of them suffered from an ecdysis phenotype and had a second cuticle like shown in Fig. 4. The remaining were very small when found dead, but no second cuticle was found. Somewhat surprisingly we also found this phenotype in *PC1/3*-RNAi larvae but at much lower penetrance. Survival of *PC1/3* larvae was 50%, and 26% of dead larvae showed the ecdysis phenotype (*n* total = 38, n of inspected dead larvae = 19), which gives a total penetrance of the phenotype of 13% (see Table 1). The failure in the moulting process and the induction of supernumerary moults in *PC1/3* RNAi indicate that the gene acts pleiotropically in multiple steps of moulting control. The results also indicate that both *PC1/3* and *PC2* act in the moult pathway and that the other protease may be able to partially rescue the process in the knockdown larvae. To test this, we screened larvae in which a double knockdown was performed (as shown in Fig. 3D) for an ecdysis phenotype. We found that 100% of these larvae (*n* = 20) suffered from this phenotype (see Table 1 for comparison), supporting the hypothesis of collaborative function in this process. In addition, we evaluated which proportion of the RNAi-larvae were able to successfully complete one or two moults (Table 1), before either dying of an ecdysis phenotype, or other causes, or surviving to the pupal stage (*PC1/3* RNAi larvae only). We found that 65% of *PC2*-RNAi larvae completed at least one moult cycle, compared to only 33% of the double knockdown larvae. None of the latter was able to complete a second moult cycle, whereas 15% of the *PC2* single knockdown were able to do so. All *PC1/3* knockdown larvae completed one moult and the large majority (89%) also completed a second moult, reflecting the low penetrance of the phenotype in this knockdown. Taken together, *PC1/3* and *PC2* act together in the moult process and can partially compensate for one another’s function while the contribution of *PC2* to this process is bigger.

**Table 1:** Phenotypes following larval RNAi

	Death as larva	Ecdysis phenotype	1 moult completed successfully	2 moults completed successfully
*dsRed* inj. (*n* = 10)	0%	0%	100%	100%
*PC1/3* RNAi (*n* = 38)	50%	13%	100%	65%
*PC2* RNAi (*n* = 20)	100%	75%	65%	15%
*PC1/3* + *PC2* RNAi (*n* = 21)	100%	100%	33%	0%

The two right-hand columns give proportions of individuals that completed 1 or 2 moults successfully (also including individuals that survived to the pupal stage (*PC1/3*-RNAi only) and individuals that died but showed no ecdysis phenotype

### Both *PC2* and *PC1/3* are essential for survival and fertility of adult beetles

**Fig. 5:**
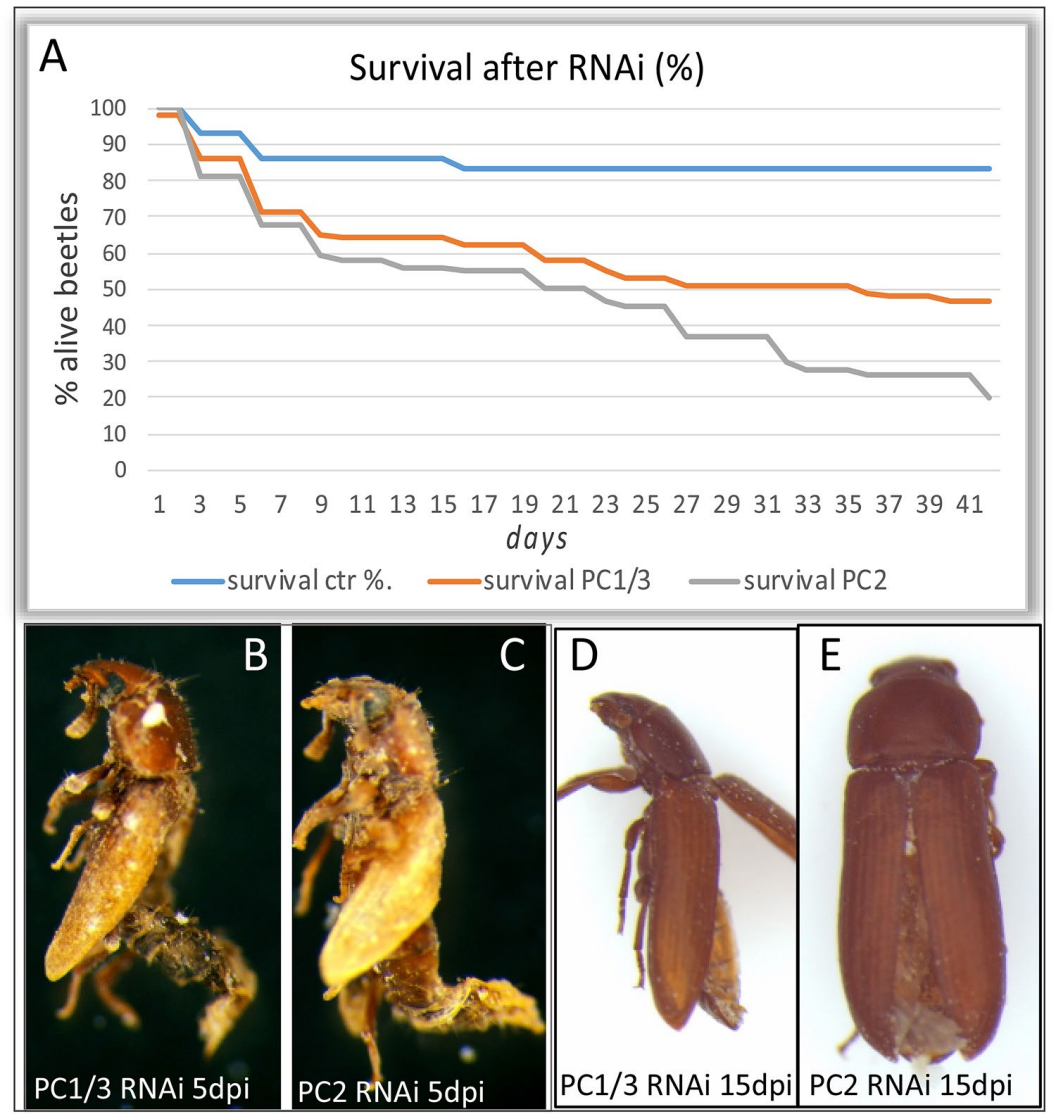
Reduced viability following RNAi targeting *PC1/3* and *PC2*. **A** Survival curves over 41 days after pupal injections, showing percentage of living beetles at the given time. Control (*dsRed*-dsRNA) *n* = 30, *PC2* and *PC1/3* *n* = 100. Note that all beetles had hatched on day 3 after injection and that deaths occurring before were likely caused by the injection process itself. **B, C** Appearance of *PC2*- and *PC1/3*-dsRNA injected pupae found dead 5 days post-injection (dpi) (lateral views). Abdomina are thin and dried out, wings are not fully elongated and not closed dorsally indicating incomplete metamorphosis. **D, E** Appearance of *PC2*- and *PC1/3*-dsRNA injected beetles found dead 15 days post-injection (dpi) (**D**: lateral view, **E**: dorsal view). Wings are fully elongated, but elytra were found fully (**D**) or half open (**E**). Abdomina were very flat and dry. Both appearances were found in either knockdown, see Table 2 for frequencies

To test the function of both genes at the adult stage we performed pupal RNAi. Female pupae where injected at a mature pupal stage: we picked pupae with pigmented eyes and sclerotised mandibles. In untreated animals eclosion occurs 1–2 days after the appearance of these features. Injections sometimes slightly delay the process. Therefore, deaths occurring up to 5 days after injection may partially be caused by injection injury (compare *dsRed* control-injected set in Fig. 5A).

We found that interfering with expression of *PC2* and *PC1/3*, respectively, led to severe reduction of survival rates compared to control-injected animals (Fig. 5A). Over the experimental time course of 41 days *PC2*-RNAi beetles (*n* = 100) had a total survival rate of only 20% whereas 48% of *PC1/3*-RNAi beetles survived, compared to 80% survival in the control (injected with *dsRed*-dsRNA, *n* = 30). A repeat experiment confirmed the reduced viability of PC1/3- and *PC2*-dsRNA injected beetles when compared to a control set: after 30 days 44% of *PC1/3*-RNAi beetles survived, 38% of *PC2*-RNAi beetles and 67% of a control set (*n* = 100/each RNAi and *n* = 15/control; see Table 2 for total frequencies of adult phenotypes).

**Table 2:** Adult phenotype frequencies following pupal RNAi

	Dead with signs of incomplete eclosion (until day 5 post-injection)	Starved/desiccated appearance (15 days post-injection)	Dead before 30 days post-injection
*dsRed* ctrl	7% (*n* = 30)	0% (*n* = 25)	22% (*n* = 45)
*PC1/3 *RNAi	14% (*n* = 100)	72% (*n* = 50)	52.5% (*n* = 200)
*PC2* RNAi	19% (*n* = 100)	80% (*n* = 50)	66% (*n* = 200)

Beetles of both knockdowns that died in the first few days after injection (up to day 5, see Fig. 5A) typically showed signs of incomplete eclosion (Fig. 5B, C, Table 2): the wings were not fully covering the abdomen. Abdomens were thin and of a dry appearance. The desiccation is possibly a result of the incomplete covering with the elytra.

Also, later in the course of the experiment, beetles with fully elongated elytra showed signs of starvation and possibly desiccation (Fig. 5D, E, also see Table 2). Wings were frequently half opened when dead, but since these beetles had gone through complete metamorphosis before, we assume that problems with metabolism and water balance led to this appearance and to their death.

**Fig. 6:**
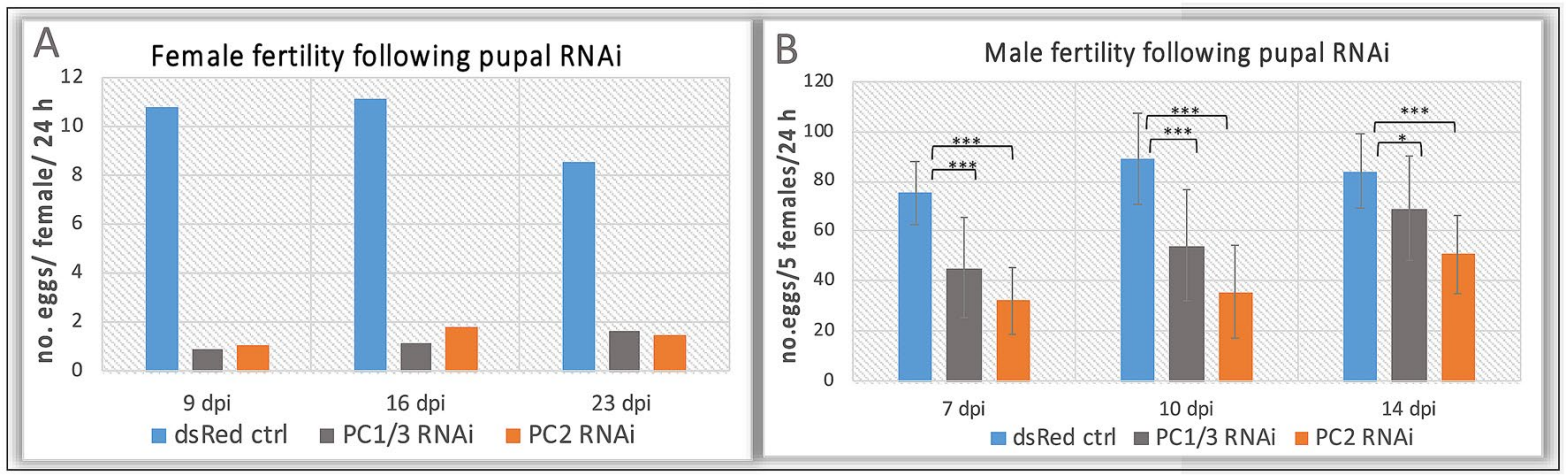
Reduced fertility in *PC1/3*- and *PC2*-RNAi beetles. **A** Both enzymes are essential for female fertility. Females (*n* = 100) were crossed to 20 males in a bulk mating. Shown are the numbers of eggs laid per female in 24 h at the given time. Number of eggs/female/24 h was > 10 in *dsRed*-dsRNA control beetles on day 9 and 16, and > 8 on day 23. In both knockdowns the number of eggs was reduced to less than 2 at all times. **B** A reduction in reproduction is observed in treated males as well. Average number of eggs laid in 24 h by 5 wild-type females that were crossed to 1 control-/RNAi-male, averaged over 30 males of each background. Error bars indicate standard deviations, T-test p-values significance levels: *** (*P* < 0.001), *(*P* < 0.05)

To test for effects on female fertility we mated females that were injected as pupae (*n* = 100 for each gene) with untreated male beetles (ratio 5:1) in a bulk mating. We assessed their reproductive success at different time points by counting all eggs in a 24-h period. We started from day 8 in order to include only adults that had successfully eclosed. We found that the number of eggs was severely reduced in both treatments. From more than 10 eggs/female/24 h egg laying decreased to less than 2 eggs/female/24 h in *PC2* and *PC1/3* knockdown animals alike (see Fig. 6A). The small number of laid eggs developed at a normal rate to wild-type appearance. An independent repetition of this experiment confirmed a lasting reduced fertility of less than 1 egg/female/24 h over 30 days, compared to 7–11 eggs/female/24 h in the control (*n* = 100/ each RNAi and *n* = 15/control, not shown).

We then tested involvement of *PC1/3* and *PC2* function in male fertility by injecting male pupae and crossing 30 individual males to wild type female beetles at a ratio of 1:5. Survival of males following injections was also reduced over the experimental time course of 14 days: *PC1/3*-RNAi: 36%, *PC2*-RNAi: 42%, *dsRed*-ctrl.: 86%. We did find significantly reduced fertility of *PC1/3* RNAi males and even more severely in *PC2* RNAi beetles (Fig. 6B). In line with a partial recovery of injected beetles from the RNAi-effect, the reduction of male fertility grew less severe the more time had passed since injection. In addition, even though egg number was reduced significantly at all three timepoints, females mated to injected males still laid a good number of eggs (Fig. 6B), and effects were generally milder than observed for female RNAi-beetles. The majority of these eggs were fertilised and developed into larvae at a rate not different from wild-type eggs (ctrl: 92% (*n* = 106), *PC1/3-*RNAi: 93% (*n* = 71), *PC2*-RNAi: 88% (*n* = 72)). These numbers indicate the presence of functioning sperm. Variation of egg number produced by individual matings was high and the death rate of the injected males was also high. The lowest number of eggs was usually obtained from males that would die shortly after. Together with the comparably moderate decrease of fertility, this suggests that the significant reduction of egg number following male treatment may have been a consequence of reduced overall fitness rather than a specific failure of sperm production or mating.

### Ovarial phenotypes underly the female infertility of *PC1/3*- and *PC2*-RNAi beetles

We aimed to further understand the causes of female infertility in *PC1/3*- and *PC2*-RNAi beetles by analysing in how far the pupal knockdown affects the ovary structure and/or oocyte maturation. In *Tribolium* the development of the telotrophic meroistic ovaries starts at the larval stage when germ cells proliferate and form 5–6 distinguishable clusters on each side. Mitotic waves of germ cells continue at the pupal stage [30]. Maturation of ovarioles and enclosure of the young oocytes with follicle cells takes place in the period from 0–5 days post adult eclosion [30, 31]. Typical number of ovarioles in adult *Tribolium* is five per side [32].

**Fig. 7:**
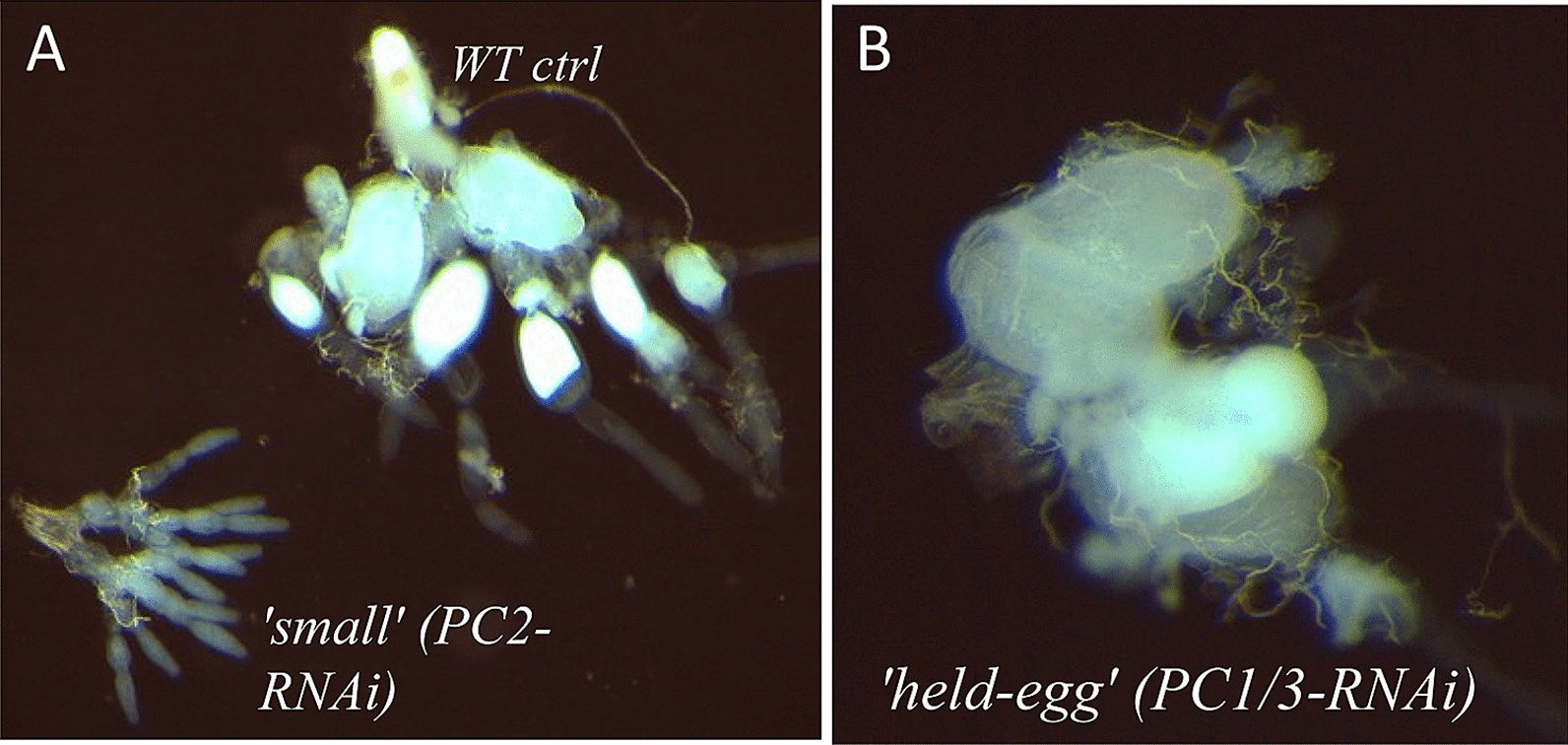
Classes of ovarial phenotypes underlying female infertility in *PC1/3*- and *PC2*-RNAi-beetles. In this analysis we distinguish two phenotypes: ‘small’ (ovaries are evidently smaller and thinner) and ‘held-egg’ (eggs accumulate in the oviduct without being laid). **A** Size comparison between a wild-type ovary and a ‘small’ ovary from a *PC2*-RNAi background. **B** Ovary displaying ‘held egg’ phenotype (from *PC1/3*-RNAi background): multiple eggs stuck in the oviducts lead to a disarranged appearance of the whole ovary. Frequencies of both these phenotypes are given in Table 3

First, we identified three classes of ovarial phenotypes (Fig. 7A, B): normal/wild-type-like, a so-called held-egg phenotype in which mature eggs accumulate in the oviduct without being laid, and a ‘small-ovary’ phenotype in which ovarioles and hence the whole ovaries are significantly smaller than the wild-type ones. We found a comparable distribution of phenotypes in both knockdowns (Table 3). Most ovaries were of the ‘small’ appearance (*PC1/3*: 62%, *PC2*: 54%), a small percentage (10% in each knockdown) were classified as ‘held-egg’, while the remaining ovaries were wild-type like.

**Table 3:** Frequencies of ovarial phenotypes following pupal RNAi

	‘Held egg’	‘Small ovaries’
Wild type (*n* = 20)	0%	0%
*dsRed *ctrl (*n* = 20)	0%	0%
*PC1/3* RNAi (*n* = 50)	10%	62%
*PC2* RNAi (*n* = 50)	10%	54%

**Fig. 8:**
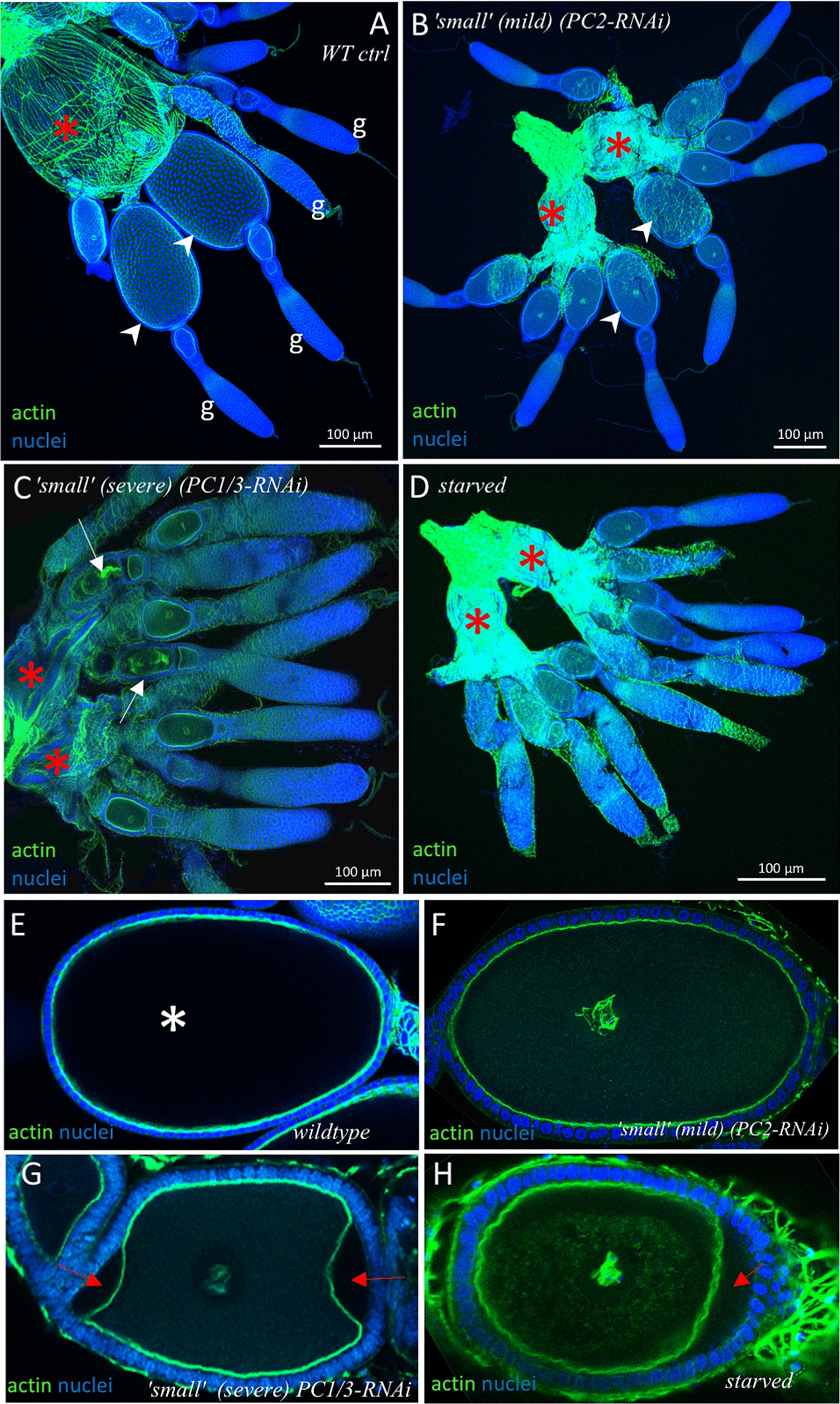
Structural analysis of ovaries using immunohistochemistry and confocal laser-scanning microscopy. **A** Wild-type ovary (one half) with germarium (marked with ‘g’) at the tips of the ovarioles and growing oocytes. White arrowheads point to oocytes that reached a mature size. Lateral oviduct (red asterisk) is widened and presumably contains mature oocytes. **B** ‘Small-ovary’ phenotype from *PC2* background: In this milder form of the phenotype the proportions of the ovarioles are maintained but the size of the most mature oocytes is reduced (white arrowheads) when compared to wild-type ovaries. Oviducts (red asterisks) do not contain mature eggs. **C** More severe ‘small-ovary’ (from *PC1/3* background). Ovarioles remain slender due to the small oocytes, which show an irregular outline. Some oocytes are seen disintegrating (white arrows) and oviducts (asterisks) do not seem to contain mature eggs. Mild and severe ‘small ovary’ phenotypes were seen in both *PC1/3* and *PC2* knockdowns. **D** Ovary of an untreated beetle starved for 10 days after eclosion, showing a similar ‘small-ovary’ morphology. Oviducts (red asterisks) do not contain mature eggs. **E–H** Higher magnification captures of individual mature oocytes. E) Wild type: the oocyte membrane lines the follicle sheet without gaps. The white star marks the expected position of the nucleus which is not stained in the fully grown wild-type oocytes, probably due to insufficient penetration of the dye. **F** Oocyte from a mild ‘small-ovary’ phenotype. Similar to wild type, the egg is oval, and the cell membrane is lined by the follicle cell sheet without gaps. **G** High magnification of an oocyte from a severe ‘small ovary’ background: The oocyte is not oval and detached from the follicle sheet (red arrows). **H** An oocyte of a ‘starved’ background displays a similar gap between the egg membrane and the follicle sheet (red arrow). **A–G** are maximum intensity Z-projections (created with imageJ) and are based on multichannel confocal laser scanning image stacks with each channel using the same plane thickness to avoid optical shift

Even though we focused on the timepoint of 14–15 days after injection, inspection of a small number of ovaries at 5 and 30 days revealed similar phenotypes. We further visualised the structure of the ‘small-ovary’ phenotype by fluorescently labelling them for actin (using phalloidin) and for nuclei (using DAPI). When comparing the ‘small ovary’ phenotype to wild-type ovaries (Fig. 8A) we found that all components of an ovariole were still present in the ‘small ovary’ phenotype (Fig. 8B, C). Like wild type, they comprise a terminal germarium followed by egg chambers with oocytes of consecutive developmental stages (mature oocyte marked by white arrowheads in Fig. 8A). In mild phenotypes the shapes of the ovarioles were not altered and the oocytes filled the whole egg-chambers, with their membrane lining the outer follicle cell sheet (Fig. 8B) like in the wild type. However, the most mature oocytes of this mild phenotype did not reach the same size as they would in wild-type ovaries (white arrowheads in 8A and B) and the lateral oviducts (red asterisks in Fig. 8A–D) were not filled with eggs as in the wild type. This indicates that these ‘small ovary’ phenotypes were not capable of producing viable eggs. The more severe form of the ‘small ovary’ phenotype (Fig. 8C) showed more slender proportions of ovarioles caused by the oocytes having a strongly reduced diameter. The oviducts are also not filled with mature eggs (red asterisks, Fig. 8C). Such defects could be primary effects of impeded neurosecretory signals or secondary due to starving of the animal. In order to distinguish these possibilities, we analysed wild-type animals, that had been starved for 4–10 days directly after eclosion. Indeed, PC knockdown ovaries looked similar to ovaries in beetles that suffered starvation (Fig. 8D, starved for 10 days, we did not find notable differences of the appearance of ovaries starved for 4, 8, 10 or 12 days, respectively). Note that severe and less severe forms of the ‘small ovary’ phenotypes were seen in both, *PC2* and *PC1/3* knockdowns. In the ‘small ovaries’ of the severe form, as in the starved ovaries, the most mature oocytes remained small and did not have the typical oval shape. There were often gaps between the follicle cells and the oocyte membrane (compare wild-type oocyte in Fig. 8E and mild form of ‘small ovaries’-oocyte in Fig. 8F to Fig. 8G and H, red arrows). We also observed a disintegration of some oocytes (white arrows in Fig. 8C), which might be followed by reabsorption of the material by the follicle cells. No mature oocytes were located in the lateral oviducts of starved ovaries (red asterisks, Fig. 8D). Intriguingly, most of the severely reduced ovaries from both knockdowns had a slightly increased number of ovarioles: *Tribolium* ovaries normally comprise 10 ovarioles whereas the severely reduced ones often had 11–12 ovarioles (see for example Fig. 8C). We also occasionally found this in ovaries from starved beetles, but never in wild-type ovaries. Due to the small number of ovaries that were fully intact after dissection and staining procedures we were not able to quantify this effect and it is also not clear how pupal RNAi or starvation from adult eclosion onwards could have caused this, given the pre-formation of ovarioles at the larval stage [30].

In summary, we found that the dominant appearance in both *PC1/3* and *PC2* knockdown was the ‘small ovary’ phenotype in which oocytes failed to reach a mature size and form. This effect could be reproduced by depriving the beetles of food, indicating insufficient availability or distribution of nutrients to the oocytes in the RNAi-beetles. Alternatively, given the specific role of a number of neuropeptides in the process of yolk deposition to the oocyte [33] this may also be a specific effect of impaired neuropeptide signalling. Note that the different classes of ovarial phenotypes taken together do not occur at a frequency that would fully explain the reduction in egg number. A proportion of 28% of *PC1/3*- and 36% of PC2-RNAi ovaries were of wild-type appearance, whereas egg number was reduced to less than 20% in both knockdowns at 16 days. Therefore, we cannot exclude additional fitness-related or behavioural effects caused by interfering with the neuroendocrine system.

## Discussion

### Phylogenetic analysis of the insect prohormone convertase family identifies *PC1/3* as evolutionarily older but less conserved

Subtilisin-like prohormone convertases trace back to unicellular organisms [7, 34] and signalling by peptide hormones is believed to be one of the earliest cell-to-cell communication system in animals [35]. We find that *PC1/3*-, *PC2*-, *PC7*-, and *furin*-orthologous genes are present in members of proto- and deuterostomes, i.e. both major bilaterian groups. Protostomes typically have *furin1* and *furin2*, whereas *PC4*, *PACE4* and *PC5* are lineage specific duplications of *furins* that are only found in deuterostomes [13]. When including sequences of the non-bilaterian species *Nematostella vectensis* into the analysis we find orthologues of *PC1/3* and *PC7*, and one *furin*, in addition to a number of PCs that do not cluster into any of the clades but predominantly form a branch by themselves (referred to as basal PCs here). This is very similar to what was found in the sponge ﻿*Amphimedon queenslandica* [13]*.* Hence, *PC1/3*, *PC7* and *furin* trace back to a pre-bilaterian origin while *PC2* is only found in Bilateria. Notably the phylogenetically younger gene *PC2* appears to be more conserved in modern invertebrate lineages whereas *PC1/3* has convergently been lost in some taxa, notably in the model organisms *Drosophila* and *C. elegans* [1, 4]. Loss of *PC7*, which, among other functions, also is involved in neurohormone processing [36] has occurred even more frequently. It is missing in all insects and in chelicerates. Interestingly, a sequence closely related to the centipede *basal PC,* and both of them related to *PC7* genes, was found in the mayfly *Cloeon dipterum*, a representative of early branching winged insects with a recently published genome [37]. Within insects, loss of *PC1/3* is restricted to the dipteran lineage only. Its absence goes in striking analogy to a reduction of neuroendocrine specific carboxypeptidases (CPs) from typically two (*CPE* and *CPD*) to only one gene in flies (*CPD*, *Drosophila: silver*) [5].

In summary, the loss of *PC1/3* and *CPE* in fly genomes makes *Drosophila* a highly derived model for this process. Similarly, the PC gene complement is highly diverged in the other protostome model system *C. elegans* [4, 38]. Given this situation, *Tribolium* represents an excellent complementary protostome model with typical gene complement and a growing toolkit for studying gene function.

### A new *PC1/3* expressing cell population of the beetle’s suboesophageal ganglion

We report a broad expression of *PC2* in the embryonic neuroectoderm and the larval CNS of *Tribolium*, which is similar to *PC2*-expression during *Drosophila* embryogenesis and in many secretory neurons of the fly nervous system [6, 39]. Expression of *PC2*-orthologues in invertebrate nervous systems and/or neuroendocrine organs has also been reported from a shrimp, a centipede, *C. elegans* and the polychaete worm *Platynereis* [38, 40–42], underlining the conserved nature of this gene.

By contrast, *PC1/3* expression or function has to date not been investigated in any invertebrate. *PC1/3* expression at the larval stage is very specific to some cells, most of which are located in the suboesophageal ganglion. Some cells of the *Drosophila* suboesophageal ganglion have a unique neuropeptide expression profile including the perivisceroscokinins *capa* and *hugin*, *fmrf*-*a* and *leucokinin* [15]. It is however yet unclear if any of these groups can potentially be homologised to the *PC1/3* cells in *Tribolium*. The presence of this specific set of cells that express *PC1/3* suggests that they have a specific role in neuroendocrine regulation. Both their developmental origin and their function require further studies.

### Essential and shared role of *PC2* and *PC1/3* in maintaining metabolic balance parallels function of both genes in vertebrates

Carbohydrate metabolism in insects is mainly regulated by two types of neuropeptides, adipokinetic hormone (AKH) and insulins. Precursors of both contain constitutive prohormone convertase cleavage sites and *Drosophila* *amon* has been shown to be required for AKH production in cells of the *corpora cardiaca* [10]. The observed poor metabolic condition of adult beetles deficient of normal *PC1/3* or *PC2* levels likely is a result of impaired AKH and/or insulin signalling. In addition, they appear to suffer from desiccation. Water balance and metabolism are closely intertwined in insects [43] and impairment of normal sugar homeostasis would impact the animals ability to retain metabolic water. In addition there are also a number of neuropeptides that control water balance by acting as diuretic hormones, such as DH31, DH37/47, and ADFs [43–45]. All these factors are likely to be affected by the knockdown of *PC1/3* and *PC2*, and we interpret the phenotype as a broad failure of multiple physiological processes, with the most prominent symptoms potentially obscuring more subtle defects.

Within vertebrates, *PC1/3* and *PC2* function is best studied in mice. Knockout of each factor leads to multiple defects in neuropeptide processing. Mutant models of *PC1/3* show high developmental and neo-natal death rates, and surviving individuals are runted [46, 47]. Similarly, the phenotype of *PC2* is reduced growth and chronic hypoglycaemia caused by a lack of glucagon and insulin processing [48–50]. In mice *PC1/3* and *PC2* partially act in the same pathways, and many neuropeptide precursors are processed by both enzymes [7]. Similar to our findings in a beetle, mutants of both enzymes show severe defects (with *PC1/3* being the more dramatic one) and there is no full redundancy between these enzymes.

### PC-dependent neuropeptide signalling is required for female oocyte maturation in a nutrient-dependent fashion and for male reproductive fitness

Most female insects deposit large amounts of nutrients, especially yolk proteins, into the developing oocyte. Our results clearly show that inhibition of both proteases disrupts this process and an insufficient amount of nutritive substances is deposited to the oocytes. This may on the one hand be caused by the inability of the injected beetles to maintain sugar and lipid homeostasis. On the other hand, neuropeptides are directly involved in regulating vitellogenesis [16]. In some insects, juvenile hormone (JH) secretion from the *corpora allata* positively regulates this process and different neuropeptides, such as allatotropin, allatostatins and ecdysis triggering hormone are involved in regulating JH-synthesis [33]. In *Tribolium* specifically, it has been shown that insulin-like peptides (ILPs), in response to JH and nutrient availability, and together with the transcription factor *Tc-FoxO*, are required for active vitellogenin transcription [51]. Hence, the severe effects on oocyte maturation that we see in *PC1/3*- and *PC2*-RNAi beetles most likely reflect physiological failures in both, the neuropeptide-dependent regulation of fat and sugar metabolism, and the specific regulated distribution of yolk proteins to the oocytes.

Male fertility and the process of spermatogenesis is far less studied than female oogenesis. Naturally the production of sperm is a process that is less nutrient intense than oocyte production. Therefore, the effect on male fecundity might be less severe because the poor metabolic condition of the beetles plays a minor role here. However, insect peptide hormones have been implicated in the regulation of testes development and sperm number [52]. In addition, neuropeptides are involved in regulating male sexual behaviour [8].

### Collaboration of both prohormone convertases in the moulting process itself with a more prominent role of *PC2* precedes redundancy of *PC1/3* in this process

The most prominent mutant phenotype of the *Drosophila PC2* homologue *amon* is developmental arrest during the first larval instar moult due to an incapability to complete ecdysis [11]. *PC2* knockdown in *Tribolium* produced the same phenotype at a high penetrance showing the conserved role of this factor in a number of involved processes. First, functional analysis of moulting fluid proteins [53] in *Tribolium* larvae frequently produced phenotypes in which cuticle shedding was impaired, similar to the prohormone convertase phenotypes we describe. Although subtilisin-like protein convertases do not appear to be abundant in the fluid [53], PC-processed neuropeptides may be involved in the initiation of moulting fluid secretion. Second, the partial digestion of the old cuticle by moulting fluid is followed by specific moulting behaviour which eventually leads to the breaking of the old cuticle [24]. Several neuropeptides have been implicated in this process. Ecdysis triggering hormone (ETH), which is secreted from the brain, starts the process. Essential for the interpretation of the ETH-signal are CCAP- (crustacean cardioactive peptide) neurons in the central nervous system [54]. Both these factors require prohormone convertase processing. Hence, failure of these two peptide signals is in theory sufficient for explaining the ecdysis phenotype. We found a minor role for *PC1/3* in the moulting process as well. *PC1/3* might contribute to the process by increasing the neuropeptide output from cells in which both proteases are co-expressed. The strong effect of *PC2*-RNAi and of the double knockdown might be explained by the interruption of the system at different sites, e.g. the subesophageal ganglion and the medial brain. In addition, either protease might be expressed in organs outside the central nervous system such as the neuroendocrine glands *corpora cardiaca* or *corpora allata*, where *Drosophila amon* (*PC2*) is expressed [6].

### *PC1/3* promotes growth and has an unexpected role in coupling growth and larval moults

With regards to larval growth, *PC1/3* and *PC2* knockdown have different effects. Following *PC1/3*-RNAi, growth is severely reduced, down to zero-growth in some individuals. *PC2*-RNAi larvae initially show a better growth rate but then undergo severe and rapid weight loss, and then die. Dramatically reduced weight is also characterising *Drosophila amontillado-* (*PC2*-) mutant larvae [12]. Inhibited growth in *PC1/3* phenotypes goes along with supernumerary moults and a prolonged larval period. Reduced growth in larvae can occur naturally by limited food supply, for example when a culture is overcrowded. It can also lead to prolonged larval development accompanied by supernumerary moults [55, 56]. Insect larvae require a species-specific critical mass to induce the hormonal cascade initiating metamorphosis [57]. In *Tribolium* metamorphosis is normally initiated when the larvae reach a weight of app. 2 mg [58]. The additional moults observed in *Tribolium PC1/3*-RNAi larvae may hence be a secondary effect of the reduced weight gain. In insects larval growth is promoted by insulin-like peptides [14, 24, 59] and the *Tribolium* genome contains a total of four *insulin-like* peptide precursor genes, which require processing to produce the mature bioactive peptides [44]. Hence, insulin-like peptides are likely candidates to be subject to *PC1/3* processing and defective *insulin* signalling may explain the reduced growth following *PC1/3* RNAi. In vertebrates pro-insulin is targeted by both *PC1/3* and *PC2* [7]. It is also possible, that the reduced growth is due to a failure of a neuropeptide regulating food intake and satiety (see [8]).

The most intriguing effect of *PC1/3* transcript depletion is that larval moults occur after minimal weight gain, and in some individuals without any positive growth in the intermoulting period. In insect larvae the non-growing cuticle limits further increase in mass and moulting is necessary to allow for additional growth. Under normal conditions the initiation of a larval moult requires some degree of growth for the larvae to reach a threshold size that triggers a moult [17, 24]. Under poor conditions larvae of many insects can switch to a size-independent mechanism and moulting can occur in response to starvation [17, 60]. Hence, reduced food uptake or impaired digestion and the resulting stalled growth in the *Tribolium PC1/3* knockdown may have led to a switch to a size-independent moulting mechanism.

The mechanism by which larvae, under non-limiting conditions, decide that growth was sufficient is not fully understood. One explanation is the hypoxia hypothesis of larval moult induction: as the tracheal system cannot not grow between moults, oxygen is suggested to become the limiting factor for the growing larva and hypoxia is then assumed to act as the trigger to induce moults [17, 61, 62]. Therefore, an alternative interpretation of the observed moults without growth following *PC1/3* knockdown in *Tribolium* is, that the processing of a neuropeptide is impaired that is involved in the yet unknown communication between the oxygen sensing factors and the moulting pathway. Whereas molecular mechanisms of hypoxia sensing are well understood in *Drosophila* [63–65], it is not known how the information of hypoxia is relayed and leads to an interpretation at the organism level [62].

## Conclusions

The independent loss of *PC1/3* in both fly and nematode genomes, might have indicated that it is rather dispensable and may function fully redundantly with its orthologue *PC2*. By contrast, our experiments showed that *PC1/3* has essential functions in the beetle. At the adult stage it works in parallel with its paralogue *PC2*, but both are not redundant as individual knockdowns cause deleterious, presumably predominantly metabolic phenotypes. The individual role of *PC1/3* in regulating growth and moulting at the larval stage is also essential, hence loss of *PC1/3* in flies is not preceded by redundancy of the factor in other insects. In line with genomic conservation, its function might well be conserved across insect groups and in flies the interplay of growth and moulting must be co-ordinated by an altered system. An analysis of *PC1/3* targets in the beetle based on cell-specific expression analysis and quantitative peptidomic approaches, together with functional testing, have the potential to characterise neuropeptide signalling systems involved in the regulation of moulting dependent on growth.

## Methods

### Genomic, phylogenetic and gene structure analysis

In order to comprehensively analyse the presence and absence of subtilisin-like protein convertases in insects we searched individual genomes of representatives of all major insect groups for which genomic information is available. We also included other invertebrate and vertebrate groups in our analysis. Genbank nr databases as well as the ENSEMBL protein database were queried between 06–2020 and 12–2020 and claimed absences were last confirmed in 05–2021 (see Additional file 1: Table S1 for all species and accession numbers). Identified sequences were classified by gene tree reconstruction using MAFFT alignment [66], and using the maximum likelihood tree reconstruction tool FastTree2 [67] with a JTT-model [68]. Lastly, we performed NCBI-Blast searches on phylum level to confirm that the detected presence-/absence- situation is typical for the whole respective insect group. To identify structural properties of the proteins the signal peptide prediction site SignalP-5.0 (CBS, Technical University of Denmark) and the domain search InterPro (EMBL-EBI) were used.

### RNA in situ hybridisation to detect gene expression

Gene fragments of *PC1/3* and *PC2* were PCR-amplified from cDNA using gene specific primers (see primer list in Additional file 1: Table S2) and inserted into a pJet1.2 cloning vector. Probes were synthesised using the 5X Megascript T7 kit (Ambion) according to the standard protocol. Embryo fixation and RNA in situ hybridisation was performed as previously described [69]. Nervous systems were dissected from mid-stage larvae and fluorescent in situ hybridisation was performed as described in [21], also including staining with an anti-Synapsin antibody (mouse, DHSB Hybridoma Bank) to visualise the neuropile of the central nervous system, and a DAPI staining for nuclei (4,6-diamidino-2-phenylindole; Sigma-Aldrich). Specimens were mounted in Vectashield (Vector labs) and a Zeiss LSM980 confocal microscope was used for image acquisition. Image stacks were analysed using FIJI [70].

### DsRNA injections and qPCR assay

Two non-overlapping fragments of each gene were PCR-amplified (see primer list in Additional file 1: Table S2) and then used for dsRNA synthesis (see Additional file 1 for details). In addition, a *dsRed*-dsRNA was generated for control injections (see Additional file 1). Quantitative PCR confirmed dsRNA efficiency and target specificity (see Additional file 1: Figure S3). For the qPCR experiments RNA samples of WT, *dsRed*-, *PC1/3*- and *PC2*-dsRNA injected larvae were isolated using a *Quick*-RNA Tissue/Insect Kit including on-column DNAseI digest (Zymo Research), and subsequently reverse transcribed to cDNA. Primer pairs were designed with at least one primer of each combination spanning a splice site (Additional file 1: Table S3) and excluding the part of the gene used for dsRNA production. Primers amplifying the ribosomal protein RPS3 were used as a standard. The qPCR experiments were run using a C1000 thermal cycler with a CFX96 detection system (Bio-Rad). See Additional file 1 for further details and see [71, 72].

All beetles used for injections and as controls were of the *San Bernadino (SB)* wild-type strain. Pupal injections were carried out as described in [20]. Larval injections were performed by first immobilising the larvae by cooling them in a dish placed on ice. They were then mounted on pre-cooled petri-dishes containing a layer of 2% agar in tab water. Lining up the larvae along an edge in the agar prevented them from moving during the injection process. Both larval and pupal injections were carried out at a 1 µg/µl concentration unless stated otherwise.

### Ovarial dissections and staining

Ovaries were dissected out of the abdomina from 14–15-day old females. A subset of ovaries classified into the respective phenotypes were further processed for fluorescent staining. The ovarioles were individually freed from their muscular lining with ultra-fine dissection needles. Ovaries were then fixed in 4% formaldehyde/ PBT for 30 min and stained overnight in Phalloidin-A488 (Sigma) at a concentration of 1:40 (v/v in PBT). Specimens were then washed 4 × 10 min in PBT and stained with DAPI (v/v 1/1000 of a 1 µg/µl stock solution in PBT) for 20 min. After 2 more PBT washes ovaries were mounted and imaged in the same way described for the stained nervous systems above.

### Survival, fertility and growth data acquisition

Following pupal RNAi, survival rates were measured by counting living individuals at 3–4-day intervals. In order to measure fertility rates all injected females (*n* = 100) were crossed with wild-type males (ratio 5:1) in a bulk mating and the total number of eggs laid in a 24-h period was counted (the number of surviving females was counted before each egg collection). The male fertility assay was set up by mating injected males to wild type females at a ratio of 1:5. Each of these male–female combinations was kept on flour in a separate petri dish. The egg number of 24-h periods was counted from each individual cross and was averaged over the total number of males used. Statistical significance was tested using 1-tailed T-test type 3 applying conventional significance thresholds of * (*P* < 0.05), ** (*P* < 0.01) and *** (*P* < 0.001).

For the generation of growth curves wild type control- and injected larvae were kept individually on 12-well plates with a non-limiting supply of flour. Larvae were checked at intervals of 2–3 days; moults were recorded, and larvae were weighed using a high precision scale (model CP225D, Sartorius).

### Larval cuticle preparations

Dead larvae were picked from the culture dishes and washed in ddH_2_O, followed by a 30 s wash in 50% bleach (Klorix) and another 2–3 washes in ddH_2_O. Specimens were then mounted in Hoyer’s medium (30 g gum arabic, 50 ml ddH_2_O, 200 g chloralhydrate, 20 mg glycerol, mixed before use with lactic acid at a ratio of 1:1) and put to 60 °C overnight, and then analysed using a standard light microscope.

## Supplementary Information

**Additional file 1.** Additional figures and tables.

## Data Availability

The datasets generated during and analysed during the current study are available in the figshare repository: https://doi.org/10.6084/m9.figshare.12981818.v1.
