# Supplementary material for: Cell-specific expression and individual function of prohormone convertase PC1/3 in Tribolium larval growth highlights major evolutionary changes between beetle and fly neuroendocrine systems
Source: EvoDevo. 2021 Jun 29;12:9. doi: 10.1186/s13227-021-00179-w (PMC8244231; doi:10.1186/s13227-021-00179-w)
Supplement: Supplementary file 1 — Additional file 1. Additional figures and tables. [file 13227_2021_179_MOESM1_ESM.pdf]

Supplementary material

1) Insect subtilisin-like proteins: gene family reconstruction

For the gene family reconstruction in insects and outgroups presented in figure 1 of the main manuscript we searched individual genomes for orthologues using vertebrate and insect pro-hormone convertases as queries. All our analysis was done on protein level and the complete list of accession number of protein sequences is given in table S1.

Table S1: Accession numbers of sequences used for gene family reconstruction. Sequences were retrieved querying the NCBI non-redundant protein database and the ENSEMBL protein database between 06-2020 and 12-2020. *partial sequences omitted from analysis, **partial sequence which is part of the same PC2-protein as XP_024081208.1, ***sequence near identical to SMAR008439-RA SKI-1, therefore omitted from alignment. ****The two *Cloeon dipterum* PC1/3 sequences may be an assembly artefact as they predominantly cover different parts of the protein and show very high sequence similarity in the overlapping area.

Species	Accession # and gene name
Nematostella vectensis	XP_032224746.1 basal PCa
	XP_032235438.1 basal PCb
	XP_032235440.1 basal PCc
	XP_001630914.2 basal PCd
	XP_001625427.2 basal PCe
	XP_032220207.1 basal PCf
	XP_032219331.1 PC7
	XP_001639811.2 PC13
	XP_032227540.1 furin
	XP_032231791.1 SKI-1
Mouse	sp|P63239.1 PC1
	sp|P21661.1| PC2
	NP_001074923.1 furin
	NP_001278113.1 PACE4
	BAA00877.1 PC4
	sp|Q61139.2 PC7
	sp|Q04592.3 PC5
	sp|Q9WTZ2.1 SKI-1
Human	AAA59918.1 PC1
	AAB32656.1 PC2
	NP_001276753.1 furin
	sp|P29122.1| PC6
	Q6UW60.2 PC4
	NP_004707.2 PC7
	NP_001177411.1 PC5
Cloeon dipterum	CAB3388312.1 PC13
	CAB3387502.1 PC13****
	CAB3364014.1 PC2
	CAB3387799.1. furin1
	CAB3362398.1 furin 2
	CAB3369459.1 basal PC
	CAB3366260.1 SKI-1
Cimex lectularius	XP_014251843.1 PC13
	XP_024081208.1 PC2
	(XP_024081896.1 PC2-like) **
	XP_014244559.1 furin1
	XP_024081814.1 furin 2
	XP_024081475.1 furin 3
	XP_014249873.1 SKI-1
Acyrthosiphon pisum	XP_008185953.1 PC13
	XP_001951256.2 PC2
	XP_008187678.1 furin1
	XP_008183106.1 furin2-1
	XP_029346915.1 furin2-2
	(XP_016662865.1 PC6) *
	XP_001952397.1 SKI-1
Apis mellifera	XP_006560472.1 PC13
	XP_392366.2 PC2
	XP_026298461.1 furin2
	XP_006562156.1 SKI-1

	Q14703.1 SKI-1
Lottia gigantea	XP_009060333.1 basal PC
	XP_009065921.1 PC7
	XP_009043850.1 PC13
	XP_009046379.1 PC2
	XP_009048793.1 furin1
	XP_009046851.1 furin2
	XP_009059034.1 SKI-1
Ixodes scapularis	XP_029851416.1 PC13
	XP_029842562.1 PC2
	XP_029839032.1 furin1
	XP_029845072 furin2
	XP_029836083.1 SKI-1
Parasteatoda tepidariorum	XP_015924123.1 PC13
	XP_015924707.1 PC2
	XP_015927741.1 furin1-1
	XP_015922741.1 furin 2
	XP_021002681.1 furin1-2
	XP_021003710.1 SKI-1
Strigamia maritima	SMAR011813-RA PC13
	SMAR004731-RA basal PC
	SMAR015491-RA PC2
	SMAR012328-RA furin1
	SMAR002108-RA furin2
	SMAR004442-RA PC7
	SMAR008439-RA SKI-1
	(SMAR003988-RA SKI-1) ***
Orchesella cincta	ODM92521.1 PC13
	ODM89572.1 PC2
	ODM97699.1 furin1
	ODM97970.1 furin2-1
	ODM92749.1 uncl. PC
	(ODM97991.1) *
Folsomia candida	XP_021968016.1 PC13
	XP_021945614 PC2
	XP_021944042.1 furin1
	XP_021943842.1 furin2
	XP_021966026.1 furin3
	XP_021958839 SKI-1
	XP_021958839 SKI-1

Pogonomyrmex barbatus	XP_011629770.1 PC13
	XP_011642625.1 PC2
	XP_011642625.1 furin1
	XP_025073722.1furin2
	XP_011631233.1 SKI-1
Bombus terrestris	XP_003400057.1 PC13
	XP_003395584.1 PC2
	XP_003398909.1 furin2
	XP_003395416.3 SKI-1
Tribolium castaneum	XP_008200350.1 PC13
	XP_972593 PC2
	XP_015840676.1 furin1
	XP_969307.1 furin2
	XP_008192186.1 SKI-1
Danaus plexippus plexippus	OWR48322.1 PC13
	OWR42183.1 PC2
	OWR47458.1 furin1
	OWR54611.1 furin 2-1
	OWR48567.1 furin 2-2
	OWR40595.1 SKI-1
Bombyx mori	XP_004925623.1 PC13
	XP_004921889.3 PC2
	XP_021205859.1 furin1
	XP_004926756.1 furin 2-1
	XP_004926747.1 furin 2-2
	XP_021206068.1 SKI-1
Drosophila melanogaster	NP_477318.1 amontillado (PC2)
	NP_001262954.1 furin1
	NP_727963.1 furin 2
	NP_649337.2 S1P (SKI-1)
Aedes aegyptii	XP_001648949.2 PC2
	XP_021709692.1 furin1
	XP_021704200.1 furin2
	XP_021705785.1 SKI-1

2) DsRNA and RNA-*in situ* probes

The two genes *PC1/3* and *PC2* share the same protein structure (figure 1C) but show very limited sequence-similarity on primary DNA level (figure S1). This allowed the production of gene specific RNA probes and dsRNA, which are unlikely to show cross-reactivity with the other paralogue.

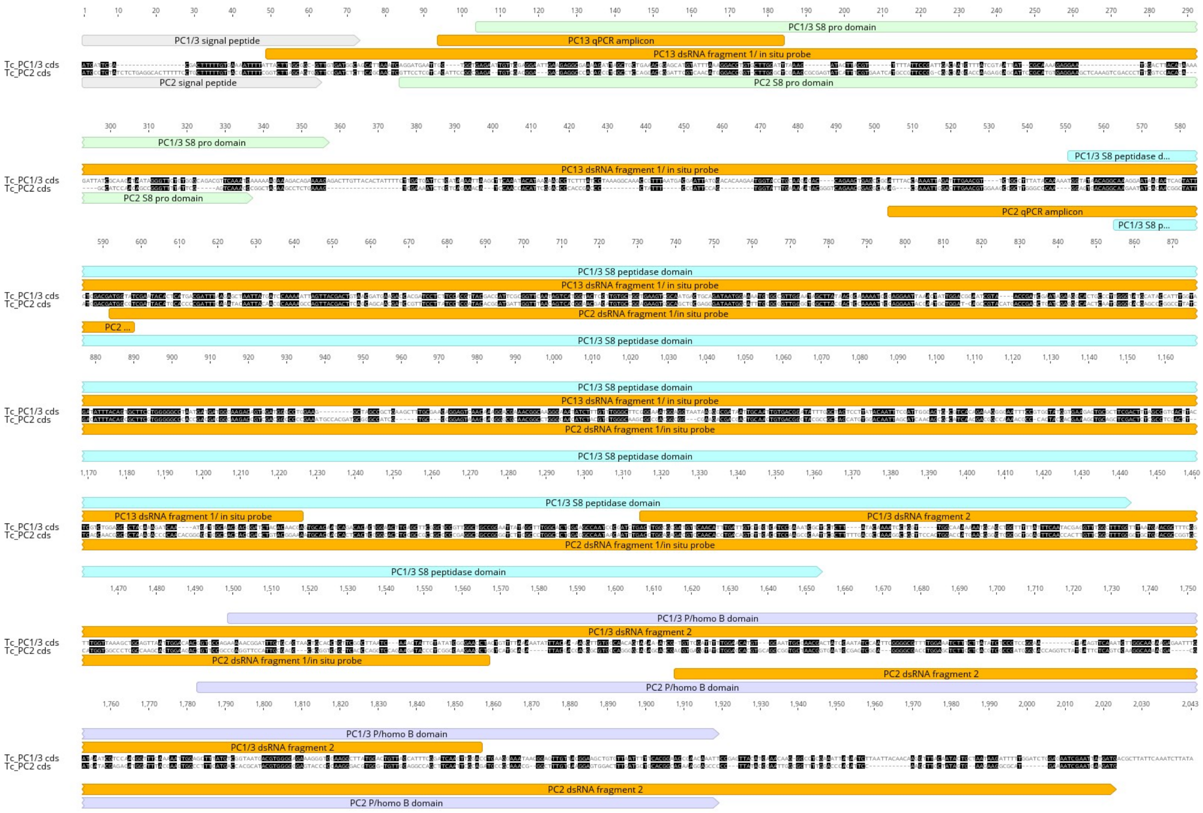

Figure S1: Alignment of *Tribolium PC1/3* and *PC2* cds DNA sequences. Alignment generated using MUSCLE [1], visualization with Geneious Prime 2021.0.1 (Biomatters). Similarities are underlined in black. Overall sequence similarity is 50.32% in the cds and the longest identical stretch is 10 bp. We have performed re-alignment of stretches with high sequence similarity (S8 peptidase domain and P/Homo B domain) but did not detect longer stretches of identical base pairs. For reference conserved domains are mapped on the alignment (compare figure 1C), as well as the PCR products used for dsRNA, in *situ probes* and qPCR amplification of each gene (compare table S2 and S3).

The primers given in table S2 were used to amplify gene sequences from cDNA for dsRNA and *in situ* probe production. Both of the two fragments were used in preliminary experiments in which they produced the same phenotypes. The experiments presented in the main paper were then carried out using fragment 2 of each gene, which does not cover the catalytic domain with a relative high sequence conservation. For the production of the dsRed dsRNA

fragment used in the control experiments a plasmid containing a 675 bp insert flanked by the SP6 and T7 promoter sites (https://www.addgene.org/68202) was used.

Table S2: Primers used for dsRNA and probe production. To amplify *PC1/3* fragment 2 we used nested PCR, with the nested product of 517 bp serving as the template for dsRNA production. For *in situ* probes targeting *PC1/3* and *PC2* fragment 1 of each gene was used.

Primer Name	Sequence	Distance
PC1/3 dsRNA fragment1 fw	TTACTTTGCGGCGTTGTGAT	1115 bp
PC1/3 dsRNA fragment1 rv	TCGTTGTGTAGATCCGTCGT	
PC1/3 dsRNA fragment2 fw	CAATCCCGATCTGACTTGGC	620 bp
PC1/3 dsRNA fragment2 rv	TTCGAGGCCCTTGTTTCATG	
PC1/3 dsRNA fragment2 nested fw	ACTTGGCGCGATGTTCAAC	517 bp
PC1/3 dsRNA fragment2 nested rv	GTCCAACTTGATCCGAAATGC	
PC2 ds RNA fragment 1 fw	ATGGCGTCGATTACATGCAC	956 bp
PC2 ds RNA fragment 1 rv	CAGCTTCTTCGCCGAGGGTA	
PC2 ds RNA fragment 2 fw	AGGTGAGGTATCTGGAGCAC	758 bp
PC2 ds RNA fragment 2 rv	AGTTTTATTTACAGTTGTCGGCC	

3) *PC1/3* RNA *in situ* hybridisation in embryos

We performed RNA *in situ* hybridisation on the full range of embryonic stages of *Tribolium* but did not detect any expression. Selected stages are shown in figure S2. Images are representative of three independent *in situ* staining experiments, each using 200-300 embryos

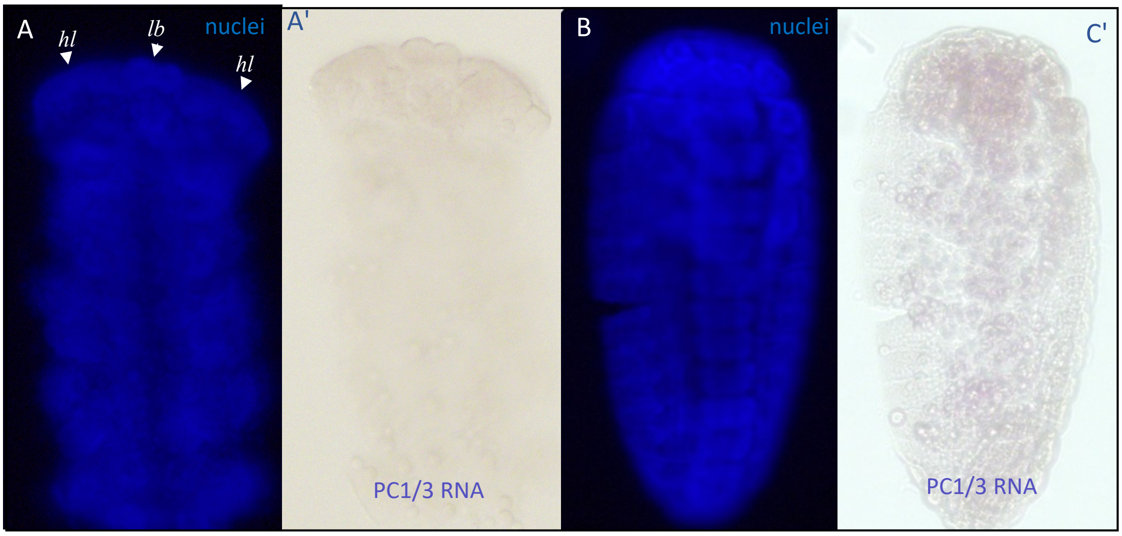

Figure S2: ***PC1/3 in situ* hybridisation in *Tribolium* embryos. A/B** showing the nuclear stain (DAPI) for staging information, **A** is a composition of two photographs of the same specimen at different focal planes fused together using photoshop CS5. **A/A'**: stage NS 10/11 (headlobes (hl) are multi-layered at this stage but the labrum (lb) is not yet fully fused but bilobed); **B/B'**: stage NS 15 (this last stage of embryonic development is clearly discernible by the oval shape of the embryo undergoing dorsal closure) Stages according to [2]. Images are representative of three independent *in situ* staining experiments, each using 200-300 embryos of the full range of embryonic stages.

4) Assessment of knockdown specificity by quantitative RT-PCR (qPCR)

To assess the efficiency of the RNAi we performed quantitative RT-PCR (qPCR). Primer pairs were tested and optimised for PCR efficiencies between 90-100 %, suitable for data analysis using the comparative Cq method [3]. Primers used are listed in table S3.

Table S3: Primers used in qPCR experiments.

Name	Sequence	Distance
PC1/3 qPCR fw	GGTGGTGAGAATTGTTGGAGG	87 bp
PC1/3 qPCR rv	AATCCAAGCACAGGTCCTTT	
PC2 qPCR fw	AGCGAAATTGGACTTGAACG	87 bp
PC2 qPCR rv	ACGCCATCGTCCATAATAGC	
RPS.3 qPCR fw	CCACTACCTGGTGGAGTTCAAG	130 bp
RPS.3 qPCR rv	GTAGTCCTCGTTGTGGGAGGT	

We tested the efficiency of our knockdowns in treated larvae 24h after injection. As a standard we used primers targeting a ribosomal proteins (RPS3) which has previously been used as a control in qPCR experiments in *Tribolium* [4]. Data were analysed using the

comparative Cq method [3]. Statistical analyses using one-way-ANOVA and Tukey Kramer test were applied to dCq values.

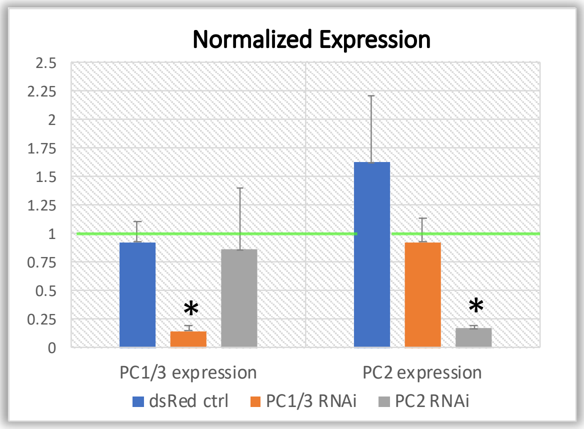

Figure S3: Normalized expression of *PC1/3* and *PC2* following RNAi. 24h post injection**.** Relative to the expression of the gene in wildtype larvae (≙1, indicated by green line). RPS3 expression was used as a standard. Values averaged over three biological replicates, error bars indicating standard deviation of expression levels between replicates. One-way-ANOVA performed on the dCq values showed significant changes between the groups (for PC1/3 expression P= 0.004165 for PC2 expression P= 0.000125). Subsequent Tukey Kramer HSD identified significant differences (abs.diff. > crit.range) between the respective targeted gene and the controls, but not between wildtype and dsRed control. There were also no significant changes between expression of the non-targeted orthologue and the controls.

Our experiments showed that RNAi levels were severely reduced in the respective knockdowns, but not in animals injected with a dsRed-dsRNA control solution. We found no cross-reactivity of *PC2* RNAi with *PC1/3* or *vice versa* that would cause the observed phenotypes. A mild and non-significant effect on the other orthologue (figure S3) may be explained by the severe overall effects of the knockdowns, potentially affecting neuroendocrine cells and networks. Additional experiments looking at later timepoints after injection found more variable knockdown levels (not shown), suggesting rescue mechanisms and/or an influence of the physiological conditions of the animals on gene expression.

5) Standard curves for larval weight gain

In an independent experiment we tested the effects of dsRNA targeting *dsRed*- a gene not present endogenously in *Tribolium*- to confirm specificity of the observed effects. We also aimed to minimize the number of controls that had to be included in the individual

experiments. Therefore, we compared the growth curves of untreated wildtype-larvae (WT) (figure S4A) to larvae injected with *dsRed*-dsRNA (n=5 of each sample) at a concentration of 2 µg/µl (figure S4B), which corresponds to the maximal dsRNA concentration used in our experiments. We also mapped the typical moult cycle to the average curves. We found that there is some variation in within the growth curves and moult cycle. The injections do however not impact the normal growth curve systematically or in a concentration dependent fashion. The majority of growth curves of control injected larvae are similar to the wildtype. The same is true for the number and the timing of the moults (figure S4 C).

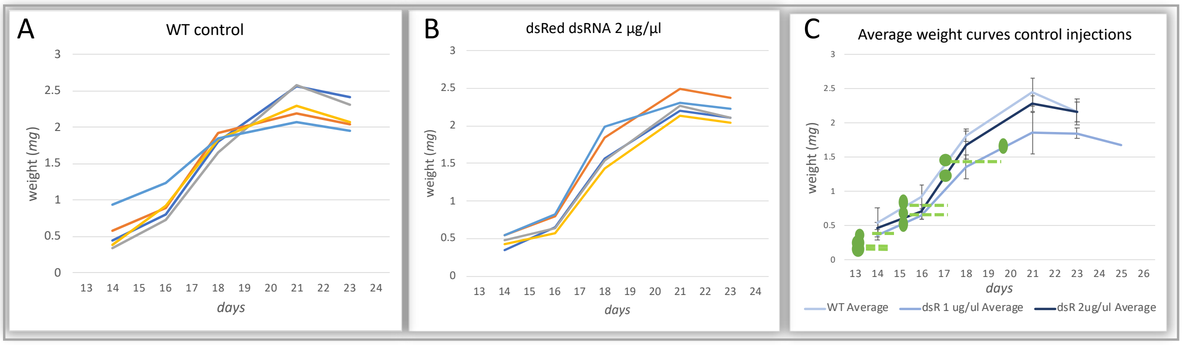

Figure S4: Standard growth curves. All larvae were isolated/injected at the age of 12 days post a 2 h egg-lay and kept individually on well plates (n=5 of each setup). The average weight of larvae on day 12 was 0.28 mg (st.d.: 0.037, n=8). Curves end at last datapoint before pupation (which in this representation can happen up to two days after the end of a curve). **A)** Individual curves of untreated WT larvae, isolated on 12-well plates at the age of 12 days, from the same batch as the injected ones. **B)** Individual curves of larvae injected with *dsRed*-dsRNA at 2 µg/µl. **C)** Average growth curves compiled from data shown in A-B, and of an additional set injected at 1 µg/µl., error bars represent standard deviations at each data point. Green cycles mark the time period between two data points in which the majority of larvae had gone through at moult, dashed green line indicates the time period during which the remaining larvae had moulted.

6) Additional experiments on larval growth following RNAi

Results from additional experiments on larval weight gain following RNAi that confirm the reproducibility of the phenotypes introduced in the main part are shown in figure S5. Larvae were injected at 14 days of age, and phenotypes were in general a bit less severe than the ones observed in larvae injected at 12 days. Nevertheless, *PC2* and *PC1/3-PC2* double RNAi larvae likewise suffered from a mortality of 100 %. Evaluation of the number of larval-to-larval moults after injection at 14 days revealed that *PC1/3* knockdown larvae that survived to the

pupal stage all went through 3 moults, compared to 2 moults in the control. Hence the increased number of moults seen in the *PC1/3* knockdown is also reproducible.

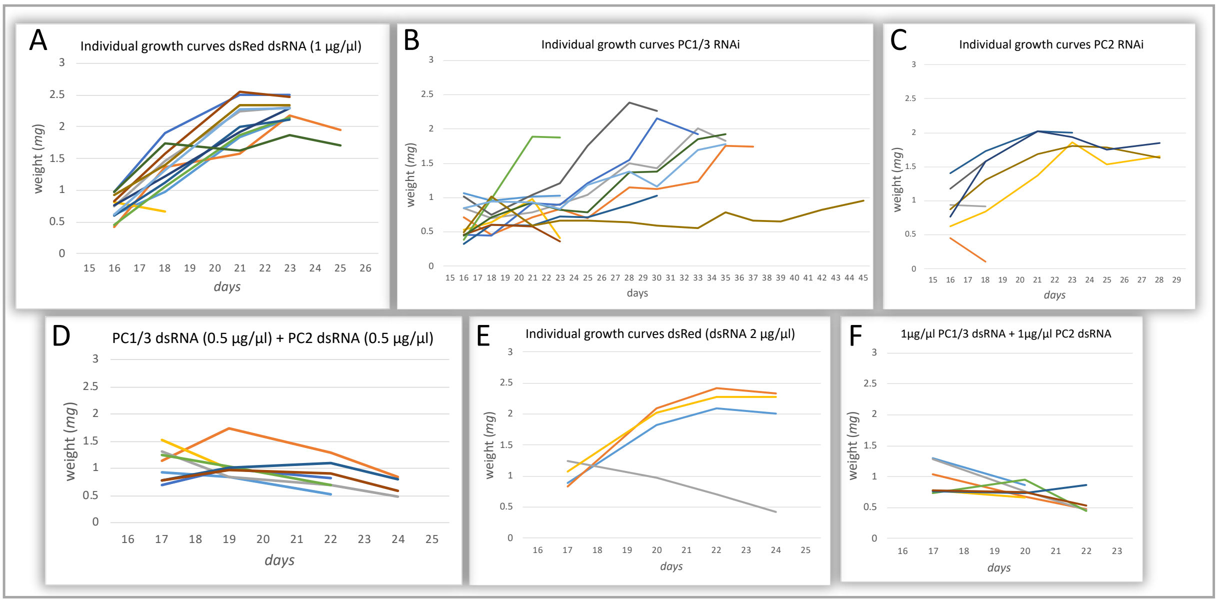

Figure S5: Larval growth curves following dsRNA injections at 14 days. All larvae were isolated/injected at the age of 14 days following a 2 h egg-lay and kept individually on well plates. Number of individuals differs between the experiments and is given below each graph. Unless stated otherwise, larvae were injected at a concentration of 1 µg/µl dsRNA. **A)** *dsRed*-dsRNAi control injected larvae, mortality: 1/13. **B)** *PC1/3*-dsRNA injected, mortality: 5/13. All larvae that survived to the pupal stage underwent three larval-to-larval moults. One larva of this experiment remained at the larval stage for 3 months, but the total number of moults during that time is unknown. **C)** PC2-dsRNA injected, mortality: 11/11. **D)** *PC1/3*-PC2 dsRNA co-injected larvae, 0,5 µg/µl each, ≙ total RNA concentration of 1 µg/µl, mortality: 8/8. **E)** *dsRed*-dsRNAi control injected larvae (2 µg/µl), mortality: 1/4 (L3). **F)** *PC1/3-PC2* dsRNA co-injected larvae, 1 µg/µl each, ≙ total RNA concentration of 2 µg/µl, mortality: 8/8.

7) Altered moult cycle in PC1/3 knockdown larvae

During our analysis of growth and moult cycle we found that growth in *PC1/3* larvae was severely slowed with some specimens showing near zero weight gain over several days. Table S4 shows the weight at specific time points and the occurrences of moults. Multiple *PC1/3* knockdown larvae (L2, L3, L4, L8) moulted one or more times despite zero weight gain, or even slightly negative growth, while other larvae moulted after minimal weight gain. This illustrates the decoupling of growth and moults in these individuals.

Table S4: Raw data on weight gain and moult cycle in *dsRed* control injected and *PC1/3*-RNAi larvae. Weight was measured in a 2-3-day rhythm and is given in mg. Yellow fields indicate that a moult has occurred between that and the previous data point. Red fields stand for dead larvae, green field mark pupal (and subsequent adult) stages. Larvae were injected at 12 days and only checked for shed cuticles but not weighed on day 13.

**dsRed-dsRNA-injected (1 µg/µl)**					**PC1/3-dsRNA-injected (1 µg/µl)**								
	specimen #					***specimen #***							
	L1	L2	L3	L4		L1	L2	L3	L4	L5	L6	L7	L8
13 days					13 days								
14 days	0.48	0.57	0.53	0.23	14 days	0.26	0.25	0.5	0.36	0.19	0.42	0.26	0.31
15 days					15 days								
16 days	0.73	0.88	0.82	0.57	16 days	0.31	0.31	0.46	0.34	0.17	0.42	0.25	0.33
17 days					17 days								
18 days					18 days								
19 days	1.5	1.51	1.74	1.25	19 days	0.36	0.31	0.55	0.32	0.18	0.43	0.27	0.33
20 days					20 days								
21 days	2.37	1.91	2.34	1.45	21 days	0.42	0.37	0.58	0.23	0.15	0.59	0.36	dead
22 days					22 days								
23 days	2.15	1.93	2.04	1.85	23 days	0.68	0.4	0.53	0.24	0.11	0.53	0.38	
24 days					24 days								
25 days					25 days								
26 days	pupa	pupa	pupa	pupa	26 days	0.64	0.34	0.51	0.24	dead	0.71	0.53	
					27 days								
					28 days	1.05	0.43	0.6	0.26		0.91	0.7	
					29 days								
					30 days	1.28	0.43	0.64	0.29		1.02	0.7	
					31 days								
					32 days								
					33 days	1.51	0.3	0.6	0.32		1.03	0.71	
					34 days								
					35 days	1.62	0.34	0.57	0.31		1.2	0.95	
					36 days								
					37 days	1.7	0.27	0.76	0.26		1.11	1.1	
					38 days								
					39 days								
					40 days	pupa	0.31	1.07	0.41		pupa	1.54	
					41 days								
					42 days		0.26	1.3	0.34			1.53	
					43 days								
					44 days		dead	1.93	0.35			1.48	
					45 days								
					46 days								
					47 days			1.77				pupa	
					48 days								
					49 days			pupa					

8) References

1. Edgar RC. MUSCLE: Multiple sequence alignment with high accuracy and high throughput. Nucleic Acids Res. 2004;32:1792–7.

2. Biffar L, Stollewerk A. Conservation and evolutionary modifications of neuroblast expression patterns in insects. Dev Biol. Elsevier; 2014;388:103–16. http://dx.doi.org/10.1016/j.ydbio.2014.01.028
3. Schmittgen TD, Livak KJ. Analyzing real-time PCR data by the comparative C T method. 2008;3:1101–8.
4. Kitzmann P, Schwirz J, Schmitt-Engel C, Bucher G. RNAi phenotypes are influenced by the genetic background of the injected strain. BMC Genomics. 2013;14.
